# QKI-induced circ_0001766 inhibits colorectal cancer progression and rapamycin resistance by miR-1203/PPP1R3C/mTOR/Myc axis

**DOI:** 10.1038/s41420-025-02478-w

**Published:** 2025-04-23

**Authors:** Yulai Zhou, Yan Gao, Yinghui Peng, Changjing Cai, Ying Han, Yihong Chen, Gongping Deng, Yanhong Ouyang, Hong Shen, Shan Zeng, Yangfeng Du, Zemin Xiao

**Affiliations:** 1 Department of Oncology, Xiangya Hospital, Central South University, Changsha, Hunan China; 2 National Clinical Research Center for Geriatric Disorders, Xiangya Hospital, Central South University, Changsha, Hunan China; 3 Department of Microbiology, Immunology & Molecular Genetics, University of Texas Long School of Medicine, UT Health Science Center, San Antonio, TX USA; 4 Department of Immunology, University of Toronto, Toronto, ON Canada; 5 Department of Emergency, Hainan General Hospital, Hainan Affiliated Hospital of Hainan Medical University, Hainan, China; 6 Department of Oncology, Changde Hospital, Xiangya School of Medicine, Central South University, Changde, Hunan China

**Keywords:** Colorectal cancer, Prognostic markers

## Abstract

Colorectal cancer (CRC) is the third most common cancer and remains a significant challenge due to high rates of drug resistance and limited therapeutic options. Circular RNAs (circRNAs) are increasingly recognized for their roles in CRC initiation, progression, and drug resistance. However, no circRNA-based therapies have yet entered clinical development, underscoring the need for comprehensive detection and mechanistic studies of circRNAs in CRC. Here, we identified and characterized a circular RNA, circ_0001766 (hsa_circ_0001766), through microarray analysis of CRC tissues. Our results showed that circ_0001766 is downregulated in CRC tissues and closely associated with patient survival and metastasis. Functional experiments demonstrated that circ_0001766 inhibits CRC cell proliferation, migration and invasion both in-vitro and in-vivo. Mechanistically, hypoxia downregulates Quaking (QKI), an RNA-binding protein essential for the biogenesis of circ_0001766 by binding to introns 1 and 3 of *PDIA4* pre-mRNA. Reduced QKI expression under hypoxic conditions leads to decreased circ_0001766 levels in CRC. Circ_0001766 acts as a competitive endogenous RNA, sponging miR-1203 to prevent the degradation of *PPP1R3C* mRNA. Loss of circ_0001766 results in decreased *PPP1R3C* expression, leading to the activation of mTOR signaling and increased phosphorylation of Myc, which promotes CRC progression and rapamycin resistance. Our study reveals that overexpression of circ_0001766 or *PPP1R3C* in CRC cells inhibits the mTOR and Myc pathway, thereby resensitizing cells to rapamycin. The combination of circ_0001766 or *PPP1R3C* with rapamycin markedly inhibits CRC cell proliferation and induces apoptosis by reducing rapamycin-induced Myc phosphorylation. In summary, our study elucidates a critical circ_0001766/miR-1203/PPP1R3C axis that modulates CRC progression and rapamycin resistance. Our findings highlight circ_0001766 as a promising therapeutic target in CRC, providing a new avenue for enhancing the efficacy of existing treatments and overcoming drug resistance.

## Introduction

Colorectal cancer (CRC) is the third most common cancer, with a 5-year overall survival rate of 64%, dropping below 15% in metastatic CRC patients [1, 2]. Despite improvements in prognosis over the past decades, therapeutic options remain largely limited to chemotherapy. A significant challenge in achieving prolonged survival with systemic anticancer therapy has been drug resistance, persisting with chemotherapy and extending to targeted therapies and immunotherapy [3]. Therefore, prioritizing mechanistic research to uncover novel therapeutic vulnerabilities in CRC is imperative.

Circular RNAs (circRNAs), a subgroup of endogenous noncoding RNAs widespread in eukaryotic cells, play significant regulatory roles in cancer initiation and progression [4, 5]. Multiple circRNAs can be formed from the same gene through mechanisms related to selective back-splicing and splicing site selection, controlled by exon skipping events and RNA-binding proteins (RBPs) such as Quaking (QKI) and Fused in Sarcoma (FUS) [6–9]. The most frequently proposed function of circRNAs is to act as competitive endogenous RNAs (ceRNAs), regulating post-transcriptional control by binding to microRNAs (miRNAs) [10–12]. While there are some controversies about circRNAs and their proposed mechanisms [13], a promising cancer therapy in preclinical studies involves delivering circRNAs with multiple miRNA-binding sites to act as ceRNAs, thereby neutralizing oncogenic miRNAs [14]. Notably, circRNAs have been implicated in essential signaling pathways that modulate therapeutic drug resistance, including resistance to cisplatin, sorafenib, and anti-PD-1 therapy [15–18]. Due to their high stability and resistance to RNA degradation, circRNAs can also serve as biomarkers for tumor diagnosis and treatment [19–21]. Thus, uncovering the precise mechanisms and functions of circRNAs can open new avenues of investigation into the initiation, metastasis, therapeutics, and drug resistance of CRC.

Hypoxia, a common and hallmark feature of most tumors, profoundly affects the progression and metastasis of CRC and influences the efficacy of chemotherapy, radiotherapy, and immunotherapy through complex mechanisms, including the mammalian target of rapamycin (mTOR) signaling pathway [22, 23]. The mTOR signaling, crucial for cell growth and survival, is dysregulated in ~30% of cancers [24–26]. Activation of the mTOR pathway can result from the loss of function of negative regulators within the signaling cascade [27]. For example, the loss of PPP2R2B leads to PDK1-dependent, but PI3K-independent, induction of Myc phosphorylation in response to the mTOR inhibitor rapamycin, resulting in rapamycin resistance [28]. Clinical interventions targeting the mTOR pathway, such as using rapamycin or modulating protein translation with pharmaceuticals, present promising avenues for chemoprevention in high-risk CRC patients [29, 30]. However, the effectiveness is limited, and predicting clinical outcomes remains uncertain [31, 32]. While long noncoding RNAs have been investigated as regulators of mTOR signaling in cancers, the role of circRNAs remains largely unclear [33]. Exploring how circRNAs interact with the mTOR pathway could unveil new therapeutic strategies and enhance the effectiveness of current treatments.

In this study, we identified a circRNA, circ_0001766 (hsa_circ_0001766), through microarray analysis, which has not been previously investigated in CRC patients. Circ_0001766 was found to be downregulated in CRC and was closely associated with the overall survival and metastasis of CRC patients. In-vitro and in-vivo functional experiments demonstrated that circ_0001766 inhibited the proliferation, migration, and invasion of CRC. Hypoxia-induced downregulation of QKI leads to the low expression of circ_0001766 in CRC. Overexpression of circ_0001766 functioned as a ceRNA, counteracting the inactivation of *PPP1R3C*, which is silenced by hsa-miR-1203 (miR-1203 is used throughout). This circ_0001766/miR-1203/PPP1R3C interaction consequently inhibited CRC progression and enhanced the therapeutic efficacy of rapamycin by inhibiting mTOR signaling and Myc phosphorylation. In summary, our findings identified a core signaling pathway revealing the mechanism of rapamycin resistance in CRC. This highlights circ_0001766 as a critical and promising therapeutic target in CRC, providing compelling experimental support for the utilization of circRNAs in CRC therapy and overcoming rapamycin resistance.

## Results

### Circ_0001766 is downregulated in CRC and correlates with positive prognosis

**Fig. 1:**
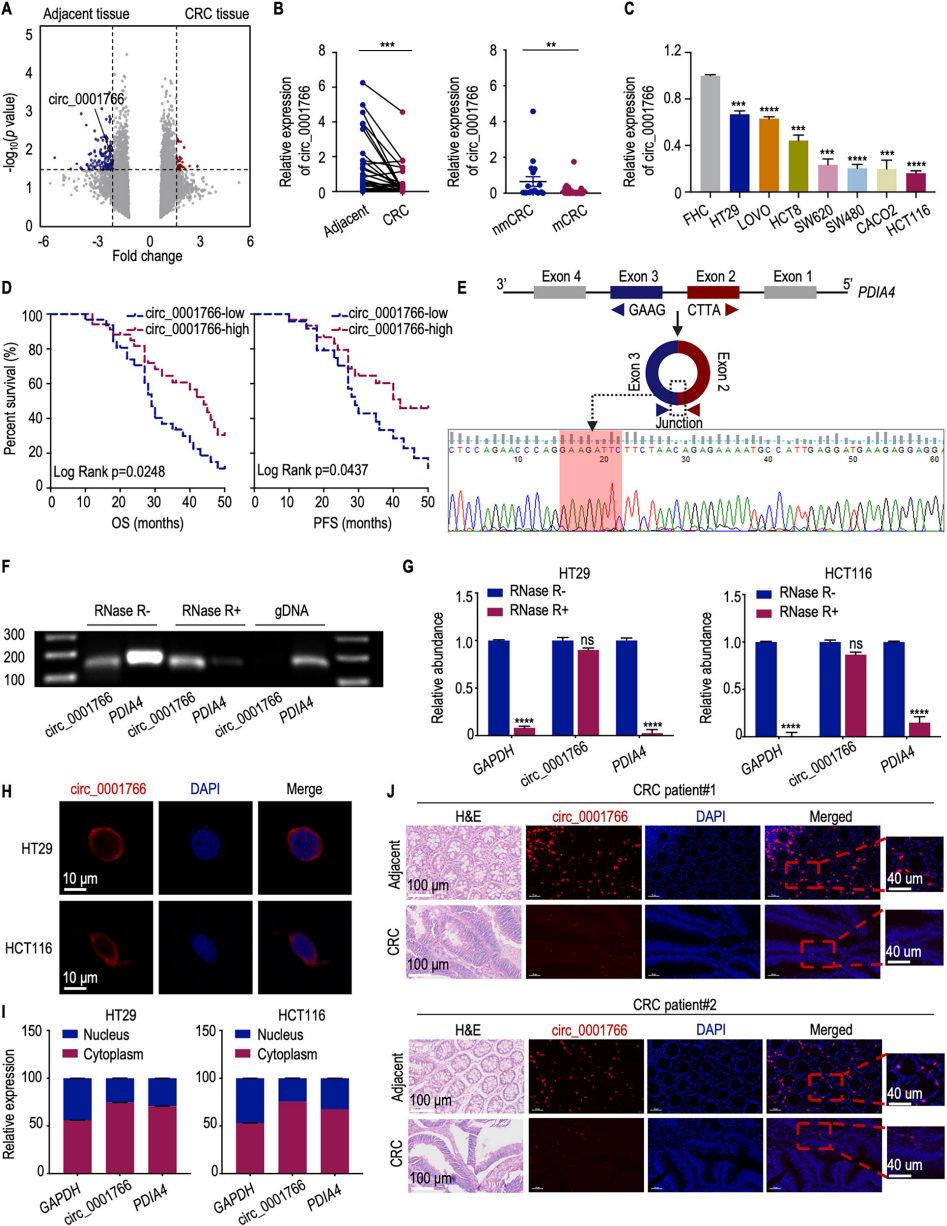
Identification of circ_0001766 in CRC tissues and cell lines. **A** Volcano plot of differentially expressed circRNAs from three pairs of CRC tissues and adjacent tissues. The −log_10_ (*P* value) is shown on the *y* axis (*P* < 0.05 indicated above-dashed line). The fold change (FC) is shown on the *x* axis (|FC | > 2 indicated left or right-dashed line). Differential expressed circRNAs are highlighted in red (upregulated in CRC tissue) or in blue (downregulated in CRC tissue). **B** (Left) qRT-PCR analyses showing the expression level of circ_0001766 in CRC tissues and paired adjacent tissues (*n* = 65). The *P* value was determined by a two-tailed paired Student *t* test. (Right) The relative expression levels of circ_0001766 in non-metastasis CRC (nmCRC) patients and metastasis CRC (mCRC) patients. The *P* value was determined by a two-tailed unpaired Student *t* test. **C** The relative expression levels of circ_0001766 in normal epithelial cell (FHC) and CRC cell lines. The *P* value was determined by a two-tailed unpaired Student *t* test. **D** Overall survival (OS) and progression-free survival (PFS) analysis in CRC stratified by circ_0001766 expression level. The log-rank test was used for survival comparison. **E** Schematic illustration showing the back splicing of circ_0001766 derived from *PDIA4* exon 2–3. The back splicing junction sequences of circ_0001766 were confirmed by Sanger sequencing. **F** RT-PCR products for detection of circ_0001766, *PDIA4* and genomic DNA (gDNA) as template in the condition with and without RNase R treatment. **G** qRT-PCR analyses for the relative expression of circ_0001766 and *PDIA4* mRNA after treating with and without RNase R in HT29 and HCT116 cells. The *P* value was determined by a two-tailed unpaired Student *t* test. **H** RNA-FISH assay showing the distribution of circ_0001766 in HT29 and HCT116 cells. Circ_0001766 probe was labeled with Cy3 (red). The nuclei were stained with DAPI (blue). Scale bar = 10 μm. **I** qRT-PCR analyses for the relative expression of circ_0001766 and *PDIA4* in the nucleus and cytoplasm of HT29 and HCT116 cells. **J** RNA-FISH analysis showing the expression and distribution of circ_0001766 in CRC tissues and paired adjacent normal tissues (*n* = 12). Tissue morphology shown by hematoxylin and eosin (H&E) staining, circ_0001766 stained with Cy3 (red) and nuclei stained with DAPI (blue). Data represent mean ± SEM. ns not significant; **P < 0.01; ***P < 0.001; ****P < 0.0001.

To delve into the precise contribution of circRNAs in CRC, we conducted microarray on 3 paired CRC tissues and adjacent normal tissues. From a total of 13,206 distinct circRNAs, we identified 156 differentially expressed circRNAs in CRC by applying a two fold change cut-off. Of these, 30 circRNAs were upregulated, while 128 were downregulated in CRC tissues compared to normal tissues (Fig. 1A). qRT-PCR analysis showed that the expression of circ_0001766 in 65 CRC tumor tissues was significantly lower compared to adjacent normal tissues. Metastatic CRC (mCRC) tissues exhibited even lower circ_0001766 expression levels compared to non-metastatic CRC (nmCRC) tissues, suggesting a potential association between circ_0001766 and CRC progression (Fig. 1B). For further investigations, HCT116 and HT29 cell lines, exhibiting the lowest and highest circ_0001766 expression levels respectively, were selected (Fig. 1C). To evaluate the clinical relevance of circ_0001766 expression, overall survival (OS) and progression-free survival (PFS) analyses were performed using the log-rank test, showing a positive correlation between higher circ_0001766 expression and better prognosis in CRC patients (Fig. 1D). Then, sanger sequencing confirmed the back-splicing site of exons 2 and 3 from the protein disulfide isomerase family A member 4 (PDIA4) pre-mRNA, validating the circular structure of circ_0001766 (Fig. 1E). Resistance to RNase R digestion supported the circular nature of circ_0001766, as linear RNAs *PDIA4* and *GAPDH* were digested by RNase R (Fig. 1F, G). Fluorescence in Situ Hybridization (FISH) visualized circ_0001766 predominantly in the cytoplasm of CRC cells (Fig. 1H). The cytoplasmic localization of circ_0001766 was further validated by nuclear-cytoplasmic fractionation assays (Fig. 1I). Notably, circ_0001766 was consistently downregulated in tissues from two CRC patients, with a predominant cytoplasmic presence (Fig. 1J). In summary, circ_0001766 is significantly downregulated in CRC, particularly in mCRC, indicating its critical role in CRC progression and metastasis.

### Circ_0001766 inhibits CRC cell proliferation, migration and invasion in-vitro and in-vivo

**Fig. 2:**
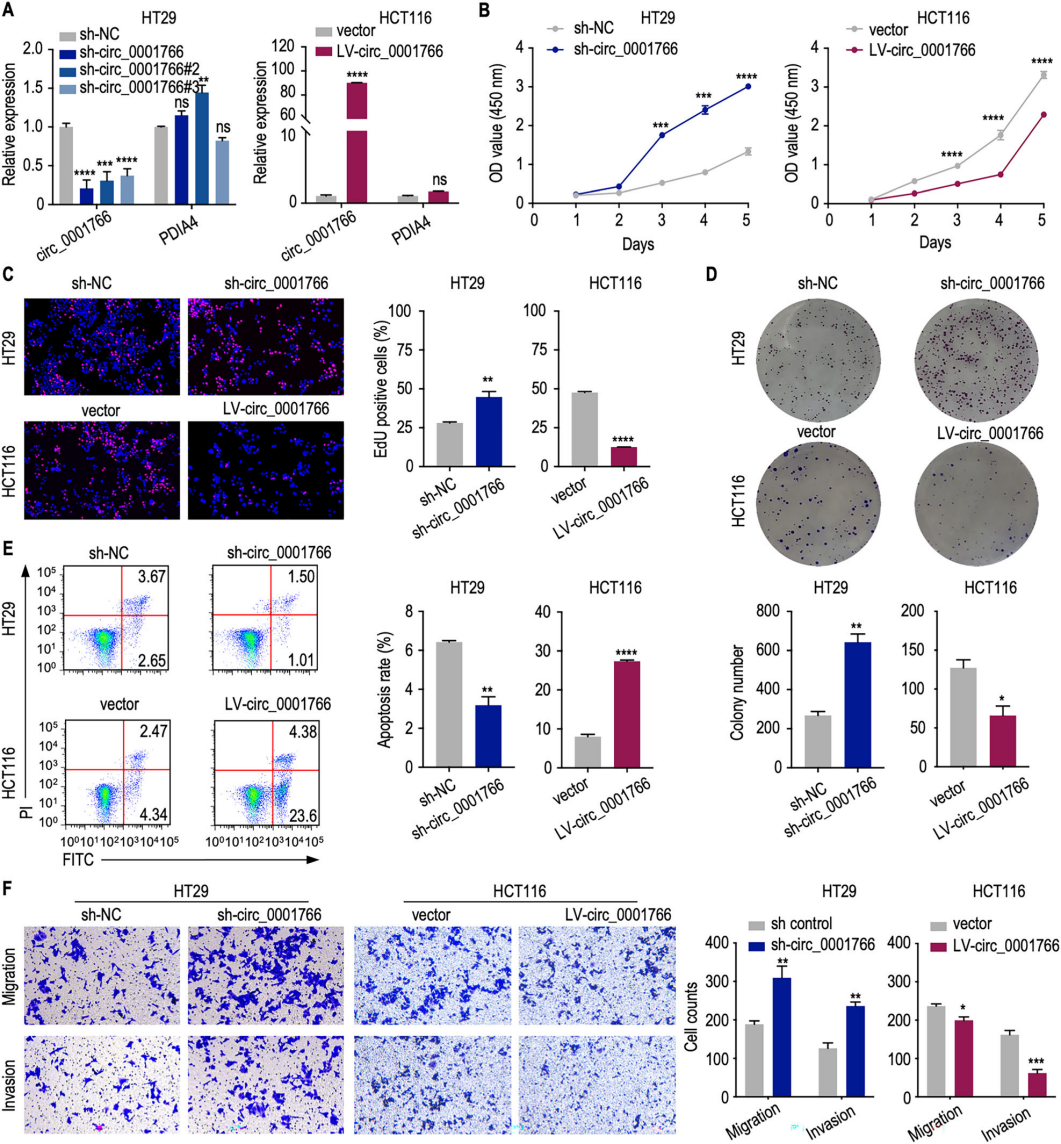
Circ_0001766 inhibited proliferation, anti-apoptosis, migration and invasion of CRC cells. **A** Relative expression of circ_0001766 and *PDIA4* in HT29 cells with control or shRNAs (left) and HCT116 cells with control and overexpressed circ_0001766 plasmid (right). The *P* value was determined by a two-tailed unpaired Student *t* test. **B**–**D** Detection of circ_0001766 inhibiting CRC cell proliferation by CCK8 assay (**B**), Edu assay (**C**), and colony formation assay (**D**). The *P* value was determined by a two-tailed unpaired Student *t* test. **E** Flow cytometry showing circ_0001766 promoted CRC cell apoptosis. The *P* value was determined by a two-tailed unpaired Student *t* test. **F** The inhibition ability of circ_0001766 for CRC cell migration and invasion was shown by transwell assay. The *P* value was determined by a two-tailed unpaired Student *t* test. Data represent mean ± SEM. ns not significant; *P < 0.05; **P < 0.01; ***P < 0.001; ****P < 0.0001.

To investigate the role of circ_0001766 in CRC cells, we employed lentivirus-packaged shRNA plasmids to stably silence circ_0001766 in HT29 cells and used lentivirus-mediated transfection to overexpress circ_0001766 in HCT116 cells. The efficiency of these transfections was confirmed via qRT-PCR (Fig. 2A). Silencing of circ_0001766 significantly increased CRC cell viability and proliferation, while overexpression of circ_0001766 showed the opposite effect, as examined by the CCK8 assay (Fig. 2B), Edu assay (Fig. 2C) and colony formation assay (Fig. 2D). Subsequently, flow cytometry analysis revealed a higher proportion of apoptotic CRC cells in the circ_0001766 overexpression group compared to controls (Fig. 2E). Silencing or overexpression of circ_0001766 profoundly influenced the migratory and invasive capabilities of CRC cells (Fig. 2F).

**Fig. 3:**
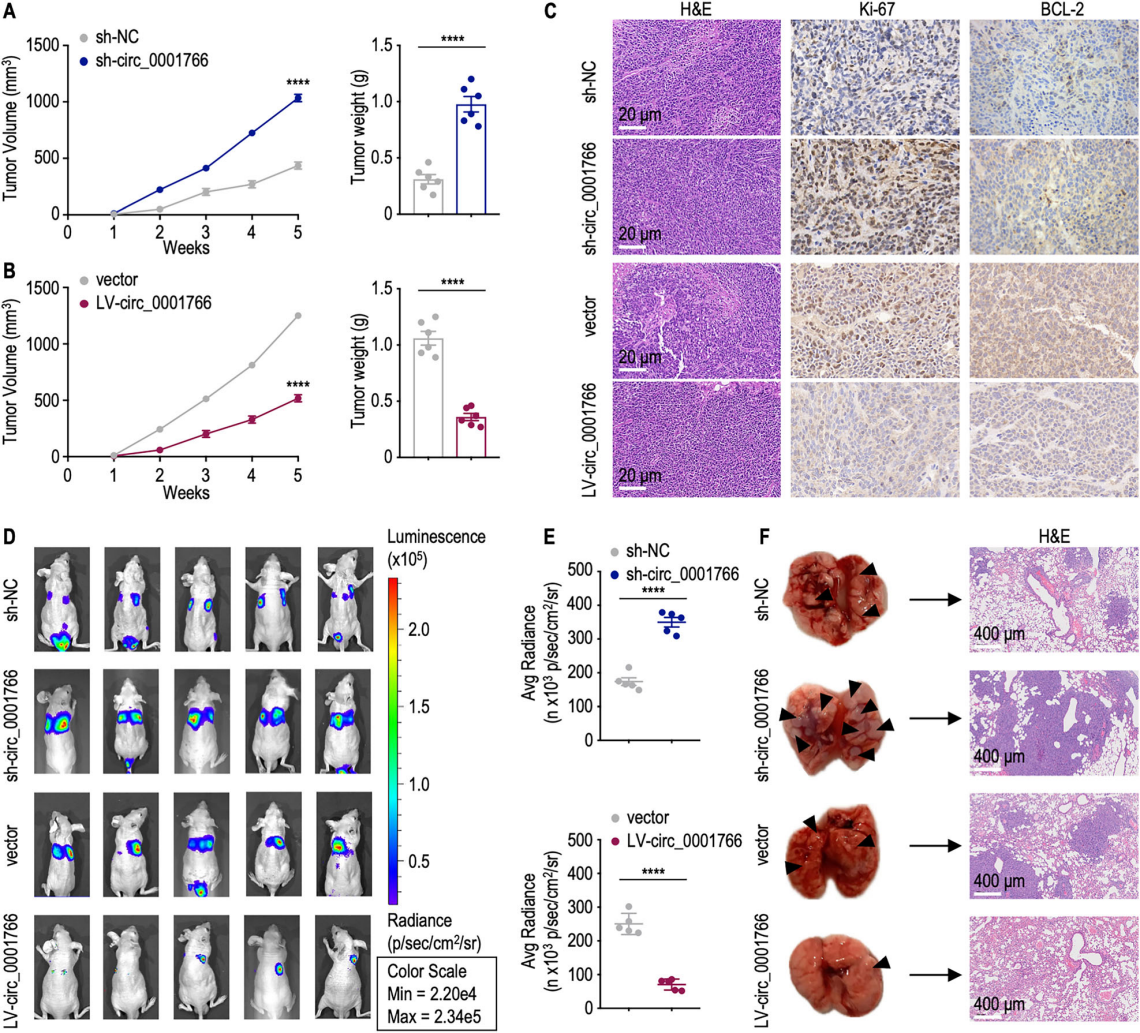
Circ_0001766 inhibited colorectal cancer growth and migration in-vivo. **A** HT29 cells transfected with control or sh-circ_0001766 were subcutaneously implanted into nude mice. Tumor volume was measured every week, and tumor weight was measured at 5 weeks after implantation (*n* = 6). Data represent mean ± SEM. The *P* value was determined by a two-tailed unpaired Student *t* test. **B** HCT116 cells transfected with vector or LV-circ_0001766 plasmid were subcutaneously implanted into nude mice. Tumor volume was measured every week, and tumor weight was measured at 5 weeks after implantation (*n* = 6). Data represent mean ± SEM. The *P* value was determined by a two-tailed unpaired Student *t* test. **C** H&E staining for tissue morphology and immunohistochemistry staining for markers of proliferation (Ki-67) and apoptosis (BCL-2) in subcutaneous tumors from mice in (**A**, **B**). Scale bars = 20 μm. **D** Luciferase activities of CRC cells were detected at day 28 after intravenous injection of HT29 cells transfected with control or sh-circ_0001766 and HCT116 cells transfected with vector or circ_0001766 plasmid (*n* = 5). **E** Average radiance intensity of lung metastatic tumor at day 28. The *P* value was determined by a two-tailed unpaired Student *t* test. **F** Representative images and H&E staining of lung metastasis tumors. Scale bars = 400 μm. Data were presented as means ± SEM. ****P < 0.0001.

We then assessed the impact of circ_0001766 on CRC cell proliferation and metastasis in-vivo. Through subcutaneous implantation and tail vein injection of sh-circ_0001766 transfected HT29 cells, circ_0001766 transfected HCT116 cells and negative control cells, the in-vivo effects of circ_0001766 on CRC growth and hematogenous metastasis in mice were determined. Overexpression of circ_0001766 significantly inhibited CRC growth, while downregulation of circ_0001766 promoted CRC growth (Fig. 3A, B). Immunohistochemical (IHC) staining using antibodies specific for the proliferation marker (Ki-67) and apoptosis markers (BCL-2) further validated the inhibitory effect of circ_0001766 on tumor progression (Fig. 3C). Using an in-vivo imaging system (IVIS), we monitored the hematogenous CRC metastasis. The tail vein model demonstrated a significant reduction in hematogenous metastasis in CRC cells overexpressing circ_0001766, whereas circ_0001766 knockdown led to increased metastasis (Fig. 3D, E). Histological examination of lung metastatic nodules through H&E staining corroborated these findings, showing a damaged pathology structure in circ_0001766 knockdown mice (Fig. 3F). Collectively, these results indicate that circ_0001766 markedly suppresses CRC cell proliferation, migration, and metastasis both in-vitro and in-vivo.

### Circ_0001766 functions by binding to miR-1203 in CRC cells

**Fig. 4:**
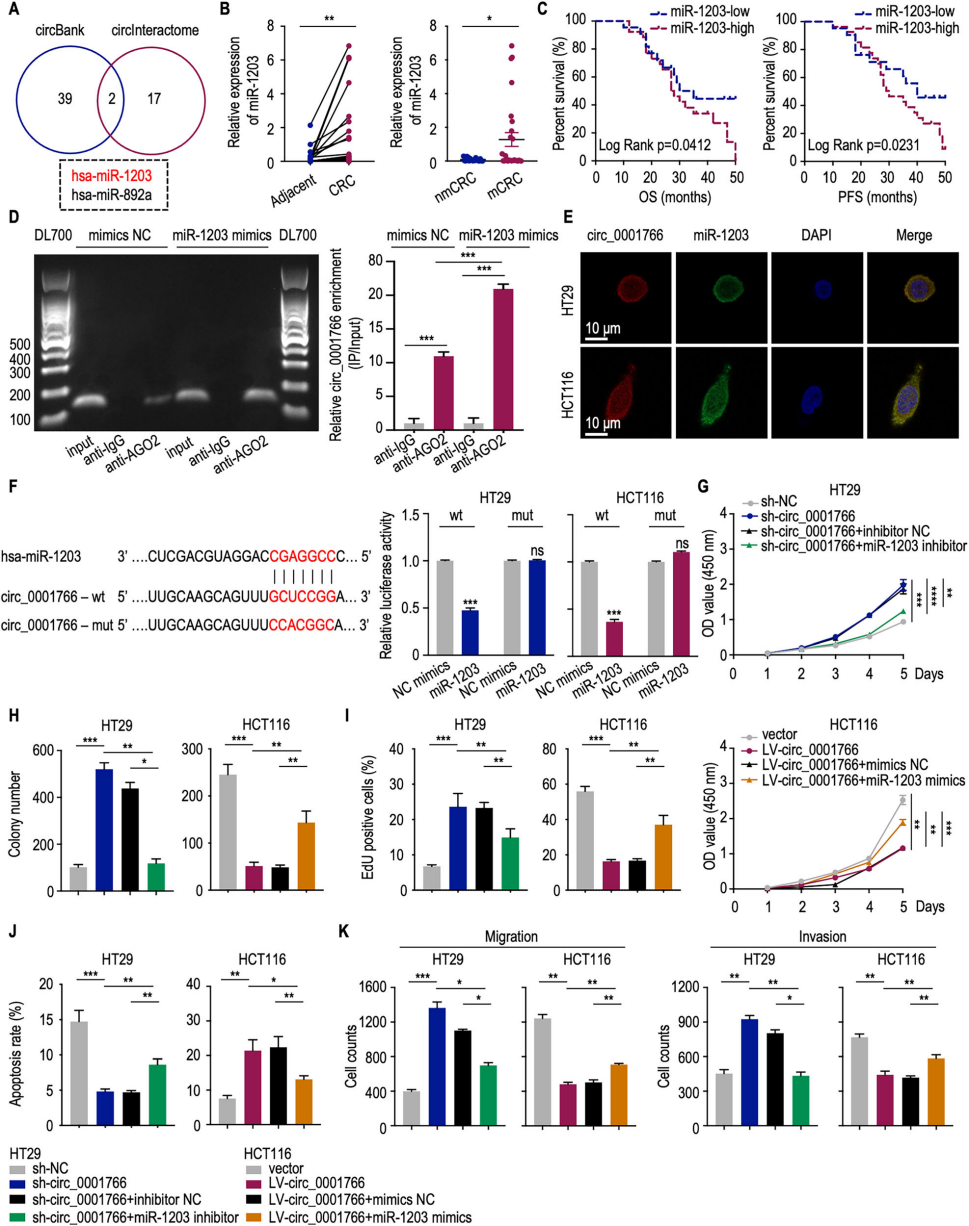
Circ_0001766 functioned as a sponge for miR-1203. **A** Venn plot showing prediction of circ_0001766 targeted miRNAs in circBank and circInteractome database. **B** (Left) qRT-PCR analyses showing the expression level of miR-1203 in CRC tissues and paired adjacent tissues (*n* = 45). The *P* value was determined by a two-tailed paired Student *t* test. (Right) The relative expression levels of miR-1203 in non-metastasis CRC (nmCRC) patients and metastasis CRC (mCRC) patients. The *P* value was determined by a two-tailed unpaired Student *t* test. **C** Overall survival (OS) and progression-free survival (PFS) analysis in CRC stratified by miR-1203 expression level. The log-rank test was used for survival comparison. **D** (Left) Agarose gel showing the binding of AGO2 and miR-1203. (Right) Quantification of miR-1203 enrichment. The *P* value was determined by a two-tailed unpaired Student *t* test. **E** RNA-FISH analysis showing the localization of circ_0001766 and miR-1203 in HT29 and HCT116 cells. Circ_0001766 stained with Cy3 (red), miR-1203 stained with FAM (green) and nuclei stained with DAPI (blue). **F** (Left) Schematic view of miR-1203 putative binding sites with circ_0001766 and construction of correspondent mutant circ_0001766 reporter plasmid. (Right) Luciferase reporter activity of circ_0001766 in HT29 and HCT116 cells co-transfected with miR-1203 mimics or NC mimics. **G**–**I** Detection of circ_0001766 inhibiting CRC cell proliferation through sponging miR-1203 by CCK8 assay (**G**), colony formation assay (**H**), and Edu assay (**I**). The *P* value was determined by a two-tailed unpaired Student *t* test. **J** Flow cytometry showing circ_0001766 promoted CRC cell apoptosis through sponging miR-1203. The *P* value was determined by a two-tailed unpaired Student *t* test. **K** The inhibition ability of circ_0001766 through sponging miR-1203 for CRC cell migration and invasion was shown by transwell assay. The *P* value was determined by a two-tailed unpaired Student *t* test. Data represent mean ± SEM. ns not significant; *P < 0.05; **P < 0.01; ***P < 0.001; ****P < 0.0001.

CircRNAs located in the cytoplasm can participate in post-transcriptional gene regulation through sponging miRNAs, thereby preventing specific miRNAs from repressing or degrading their target mRNAs [10]. Using circBank [34] and circInteractome [35] database, we identified miR-1203 and miR-892a as potential miRNAs targeted by circ_0001766 (Fig. 4A). While miR-892a has been reported to promote CRC cell proliferation through regulating *PPP2R2A* expression, miR-1203 has yet to be explored in CRC [36]. qRT-PCR analysis revealed that miR-1203 was highly expressed in CRC cell lines and tissues compared to adjacent normal tissues, with even higher expression in mCRCs than in nmCRCs (Figs. 4B and S1A). The expression of circ_0001766 was negatively correlated with miR-1203, suggesting the potential of circ_0001766 acting as a sponge for miR-1203 in CRC (Fig. S1B). OS and PFS analyses using log-rank method showed a negative correlation between miR-1203 expression and prognosis in CRC patients (Fig. 4C). AGO2 protein is an effector in RNA interference binding to miRNAs and triggering degradation of target transcripts [37]. We then performed RIP assay with an anti-AGO2 antibody showing a significant enrichment of circ_0001766 following the transfection of miR-1203 mimics into HCT116 cells (Fig. 4D). Additionally, FISH assay demonstrated the colocalization of circ_0001766 and miR-1203 in the cytoplasm (Fig. 4E). Using a circ_0001766 mutant luciferase reporter plasmid, we observed that the luciferase activity of the wildtype circ_0001766 reporter was significantly reduced compared to the mutant reporter in both HT29 and HCT116 cells (Fig. 4F). These results confirm that circ_0001766 acts as a sponge for miR-1203 in CRC cells.

To validate the interaction between circ_0001766 and miR-1203 in CRC cells, we transfected miR-1203 inhibitors into HT29 cells and miR-1203 mimics into HCT116 cells. The efficiency of these transfections was confirmed by qRT-PCR (Fig. S1C). Inhibiting miR-1203 in circ_0001766-knockdown HT29 cells reversed the increased cell viability and proliferation, while introducing miR-1203 mimics into circ_0001766-overexpressing HCT116 cells impaired circ_0001766’s function. This was examined by CCK8 assay (Fig. 4G), colony formation assay (Figs. 4H and S1D) and Edu assay (Figs. 4I and S1E). Finally, the anti-apoptotic, migratory, and invasive capabilities of CRC cells affected by circ_0001766 were also reversed by miR-1203 mimics (Figs. 4J, K and S1F, G). Thus, circ_0001766 inhibits CRC cell proliferation, anti-apoptosis, migration and invasion by sponging miR-1203.

### Circ_0001766 inhibits CRC cells via miR-1203/PPP1R3C axis

**Fig. 5:**
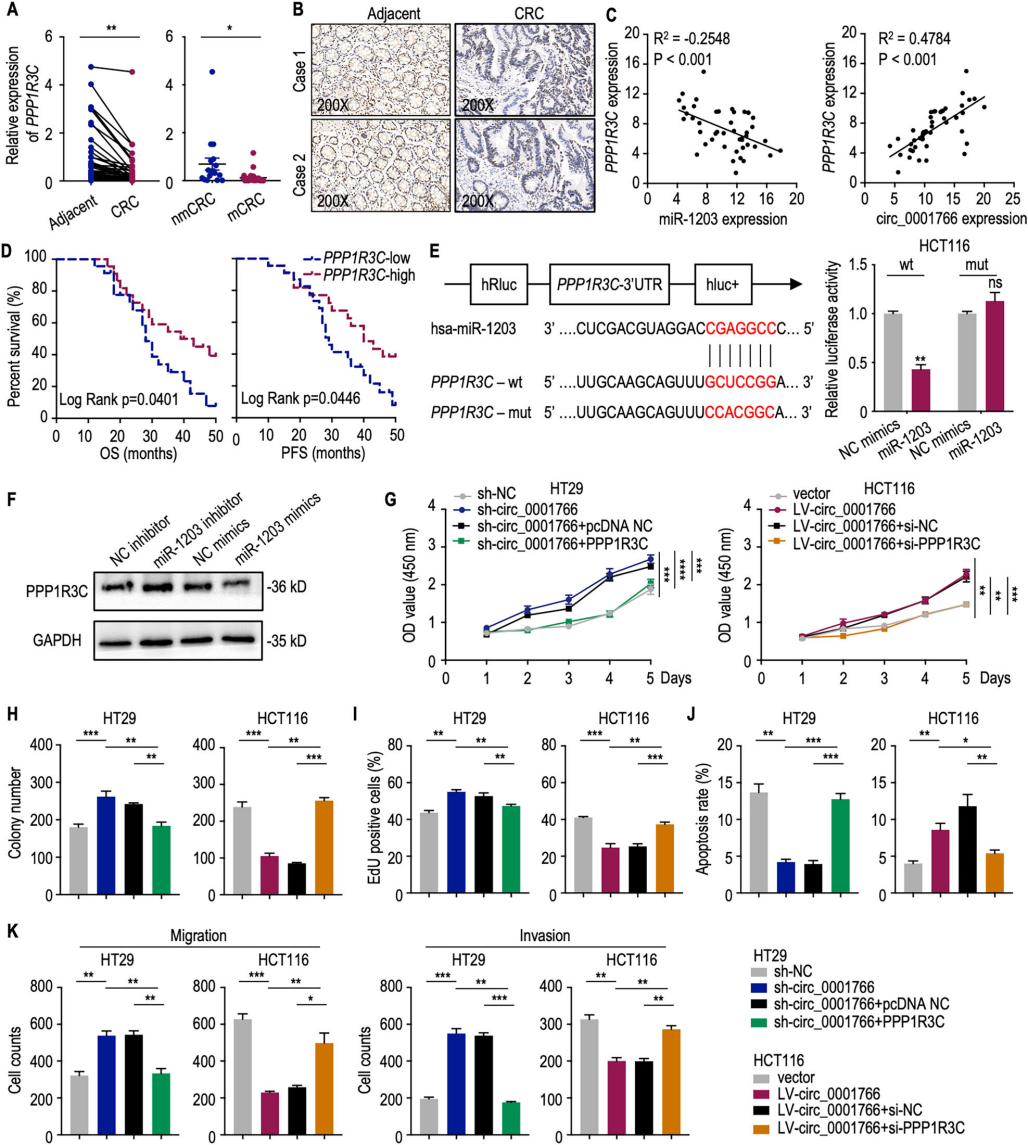
Circ_0001766 functioned through miR-1203/PPP1R3C axis. **A** (Left) qRT-PCR analyses showing the expression level of *PPP1R3C* in CRC tissues and paired adjacent tissues (*n* = 45). The *P* value was determined by a two-tailed paired Student *t* test. (Right) The relative expression levels of *PPP1R3C* in non-metastasis CRC (nmCRC) patients and metastasis CRC (mCRC) patients. The *P* value was determined by a two-tailed unpaired Student *t* test. **B** IHC staining of PPP1R3C in CRC patient adjacent tissues and CRC tissues (*n* = 12). **C** The Pearson correlation analysis between circ_0001766 and *PPP1R3C* expression in CRC tissues. **D** Overall survival (OS) and progression-free survival (PFS) analysis in CRC stratified by *PPP1R3C* expression level. The log-rank test was used for survival comparison. **E** (Left) Schematic view of miR-1203 putative binding sites with *PPP1R3C* and construction of correspondent mutant *PPP1R3C* reporter plasmid. (Right) Luciferase reporter activity of *PPP1R3C* in HCT116 cells co-transfected with miR-1203 mimics or NC mimics. **F** Western blot of PPP1R3C protein in HCT116 cell transfected with control, miR-1203 inhibitor and miR-1203 mimics. **G**–**I** Detection of circ_0001766 inhibiting CRC cell proliferation through increasing expression of *PPP1R3C* by CCK8 assay (**G**), colony formation assay (**H**), and Edu assay (**I**). The *P* value was determined by a two-tailed unpaired Student *t* test. **J** Flow cytometry showing circ_0001766 promoted CRC cell apoptosis through PPP1R3C. The *P* value was determined by a two-tailed unpaired Student *t* test. **K** The inhibition ability of circ_0001766 through PPP1R3C for CRC cell migration and invasion was shown by transwell assay. The *P* value was determined by a two-tailed unpaired Student *t* test. Data represent mean ± SEM. *P < 0.05; **P < 0.01; ***P < 0.001; ****P < 0.0001.

To investigate the target genes of miR-1203, we integrated data from miRDB [38], Targetscan [39, 40] and miRwalk [41] databases, resulting in a list of 16 overlapping genes (Fig. S2A). Analyzing gene expression and prognostic value in TCGA-COAD, we found that *TCMM40L*, *SLC8A1*, *PPP1R3C*, *P2RX1* and *HMX2* were significantly downregulated in cancer tissues (Fig. S2B). Among these, *PPP1R3C* was the only gene showing significant overall survival prognosis value (Fig. S2C, D). More importantly, *PPP1R3C* expression was downregulated in HCT116 cells transfected with miR-1203 mimics, whereas the expression of *TCMM40L*, *SLC8A1*, *P2RX1* and *HMX2* remained unchanged (Fig. S2E). The downregulated expression of *PPP1R3C* was further verified in CRC cells and tissues, especially in mCRCs (Figs. 5A and S2F). IHC from two CRC patients also validated the lower protein expression of PPP1R3C in CRC than in adjacent normal tissues (Fig. 5B). Pearson correlation analysis revealed that *PPP1R3C* expression was positively correlated with circ_0001766 expression while negatively correlated with miR-1203 expression in CRC tissues (Fig. 5C). OS and PFS analyses using log-rank method showed a positive correlation between *PPP1R3C* expression and prognosis in CRC patients (Fig. 5D). Dual-luciferase reporter assay demonstrated that the luciferase activity of the wildtype *PPP1R3C* reporter was significantly reduced compared to the mutant reporter in HCT116 cells (Fig. 5E). Western blots further indicated that miR-1203 inhibitors enhanced PPP1R3C protein levels, while miR-1203 mimics reduced PPP1R3C protein levels (Fig. 5F). These results confirm that miR-1203 can bind to *PPP1R3C*, leading to decreased PPP1R3C levels.

To verify the interactions among circ_0001766, miR-1203 and *PPP1R3C* in CRC cells, we transfected *PPP1R3C* overexpressed plasmid into HT29 cells and si-*PPP1R3C* into HCT116 cells, respectively. qRT-PCR and western blots were applied to validate the efficiency of transfection (Fig. S2G, H). Increasing *PPP1R3C* expression in circ_0001766 knockdown HT29 cells rescued the increased cell viability and proliferation, while inhibiting *PPP1R3C* in circ_0001766 overexpressing HCT116 cells impaired the function of circ_0001766, as examined by the CCK8 assay (Fig. 5G), colony formation assay (Figs. 5H and S3A) and Edu assay (Figs. 5I and S3B). Furthermore, the anti-apoptotic, migratory, and invasive effects of circ_0001766 in CRC cells were also reversed by silencing PPP1R3C (Figs. 5J, K and S3C, D). Thus, circ_0001766 acts as a sponge for miR-1203, increasing the expression of PPP1R3C, which leads to the inhibition of CRC cell proliferation, anti-apoptosis, migration, and invasion.

### Circ_0001766/PPP1R3C overexpression diminishes rapamycin-induced p-Myc and resensitizes CRC cells to rapamycin

**Fig. 6:**
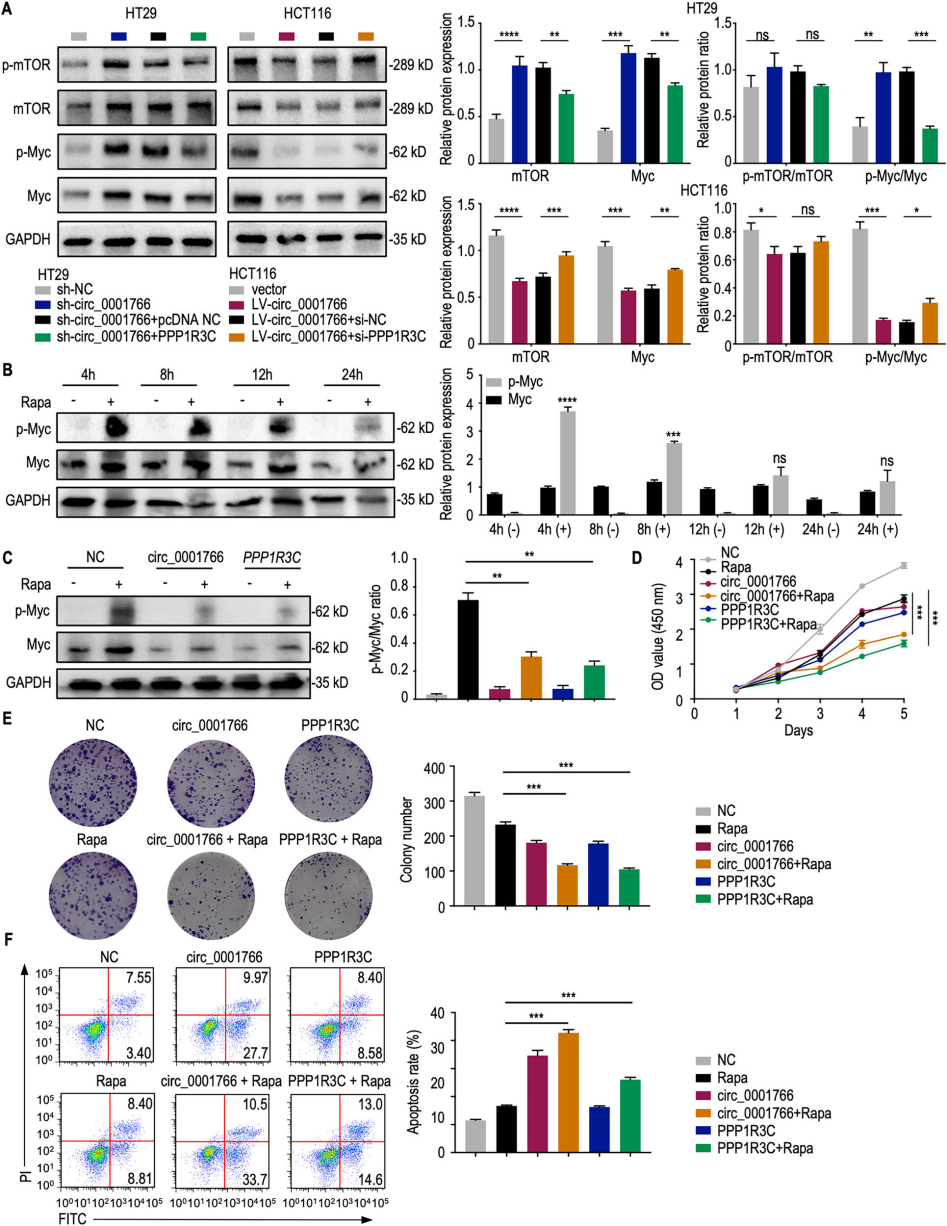
Restoration of circ_0001766 or PPP1R3C abrogated rapamycin-induced Myc phosphorylation and resensitized CRC cells to rapamycin. **A** (Left) Western blot of indicated protein in HT29 and HCT116 cells. (Right) Quantification of relative protein expression or ratio depicted as histograms. The *P* value was determined by a two-tailed unpaired Student *t* test. **B** (Left) Western blot of p-Myc/Myc protein in HCT116 cells treated with 10 nM rapamycin for the indicated times. (Right) Quantification of relative protein expression depicted as histograms. The *P* value was determined by a two-tailed unpaired Student *t* test. **C** (Left) Western blot of Myc/p-Myc protein in HCT116 cells treated with or without 10 nM rapamycin and transfected with or without circ_0001766 and PPP1R3C. (Right) Quantification of relative protein expression depicted as histograms. The *P* value was determined by a two-tailed unpaired Student *t* test. **D**, **E** Detection of circ_0001766 and PPP1R3C overexpression inhibiting CRC cell proliferation and resensitizing rapamycin by CCK8 assay (**D**) and colony formation assay (**E**). The *P* value was determined by a two-tailed unpaired Student *t* test. **F** Flow cytometry showing circ_0001766 and PPP1R3C overexpression promoted CRC cell apoptosis and rapamycin sensitivity. The *P* value was determined by a two-tailed unpaired Student *t* test. Data represent mean ± SEM. ns not significant; *P < 0.05; **P < 0.01; ***P < 0.001; ****P < 0.0001.

Protein phosphatase 1 (PP1) and protein phosphatase 2A (PP2A) are the most abundant serine/threonine phosphatases [42]. PPP2R2B has been reported to control Myc phosphorylation and modulate rapamycin sensitivity in CRC [28]. We explored whether PPP1R3C has a similar function. Indeed, silencing circ_0001766 led to increased mTOR and Myc protein levels, as well as Myc phosphorylation in HT29 cells. Overexpressing PPP1R3C counteracted this effect. Conversely, overexpressing circ_0001766 reduced mTOR and Myc protein levels and inhibited Myc phosphorylation in HCT116 cells, while silencing PPP1R3C reversed this effect (Fig. 6A). Therefore, circ_0001766/miR-1203/PPP1R3C axis affected mTOR and Myc protein levels, as well as Myc protein phosphorylation, in CRC cells.

Rapamycin triggers a compensatory Myc phosphorylation, resulting in resistance [28]. A time-course analysis showed that the Myc response to rapamycin occurred as early as 4 h, verifying the compensatory event in response to mTOR inhibition (Fig. 6B). Importantly, both circ_0001766 and PPP1R3C overexpression reduced p-Myc/Myc ratio when treated with rapamycin in HCT116 cells, indicating a suppression role of rapamycin resistance (Fig. 6C). Further, we verified that overexpression of circ_0001766 and PPP1R3C, combined with rapamycin treatment, inhibited CRC cell viability and proliferation more effectively than rapamycin alone. This was demonstrated using CCK8 assays (Fig. 6D), colony formation assays (Fig. 6E), and apoptosis flow cytometry (Fig. 6F). Thus, our data reveal an important pathway underlying rapamycin resistance in CRC, dependent on the circ_0001766/miR-1203/PPP1R3C/Myc axis.

### Circ_0001766 is inactivated due to hypoxia induced downregulation of QKI in CRC

**Fig. 7:**
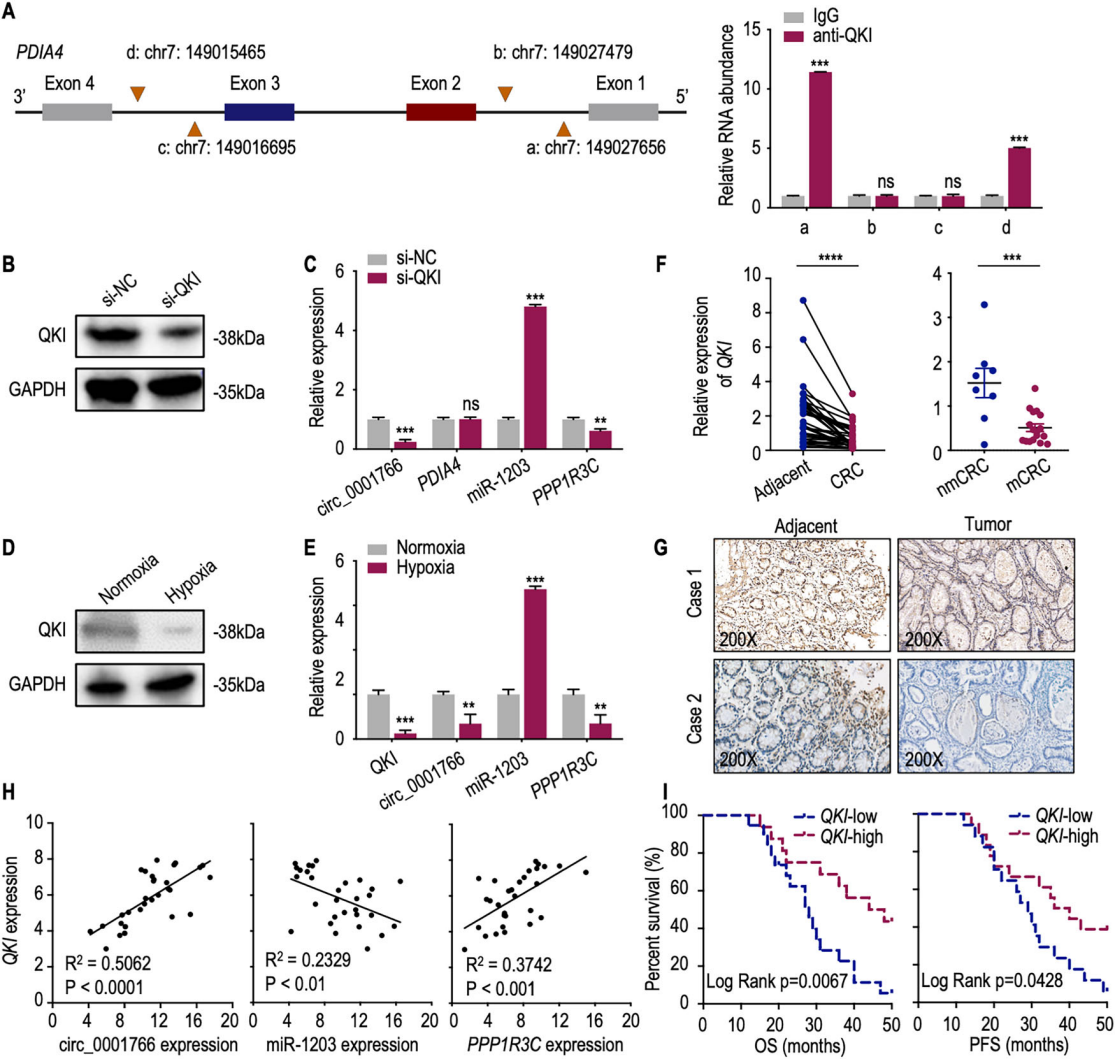
Downregulation of QKI under hypoxia microenvironment impaired the generation of circ_0001766 in CRC cells. **A** (Left) A schematic view showing the predicted QKI binding site in introns 1 and 3 of PDIA4 pre-mRNA. (Right) RIP assays showing the RNA enrichment in PDIA4 intron 1 and intron 3 QKI binding sites in HCT116 cells. The *P* value was determined by a two-tailed unpaired Student *t* test. **B** The protein levels of QKI in HCT116 cells silencing with siRNA targeting *QKI*. **C** The expression levels of circ_0001766, *PDIA4*, miR-1203 and *PPP1R3C* in HCT116 cells transfected with si-QKI. The *P* value was determined by a two-tailed unpaired Student *t* test. **D** Western blot of QKI protein in HCT116 cells under normoxia and hypoxia. **E** The expression levels of *QKI*, circ_0001766, miR-1203 and *PPP1R3C* in HCT116 cells under normoxia and hypoxia. The *P* value was determined by a two-tailed unpaired Student *t* test. **F** (Left) qRT-PCR analyses showing the expression level of *QKI* in CRC tissues and paired adjacent tissues (*n* = 45). The *P* value was determined by a two-tailed paired Student *t* test. (Right) The relative expression levels of *QKI* in non-metastasis CRC (nmCRC) patients and metastasis CRC (mCRC) patients. The *P* value was determined by a two-tailed unpaired Student *t* test. **G** IHC staining of QKI in CRC patient adjacent tissue and CRC tissue (*n* = 12). **H** The Pearson correlation analysis between *QKI* expression and circ_0001766 (left), miR-1203 (middle), and *PPP1R3C* (right) expression in CRC tissues. **I** The CRC patients with higher *QKI* expression had a higher percent of overall survival (OS) compared with the patients with lower *QKI* expression. The log-rank test was used for survival comparison. Data represent mean ± SEM. ns not significant; ***P < 0.001.

RBPs widely participate in the regulation of circRNA formation [7, 8, 43, 44]. Therefore, we predicted which RBP is involved in the regulation of circ_0001766 production through RBPmap [45]. Among these RBPs, QKI was identified as the only protein reported to generate another circRNA by binding to *PDIA4* introns 2 and 4 in gastric cancer cells [46]. Given that human *PDIA4* exon 2 and 3 circularization forms circ_0001766, we chose the top two significant binding sites to verify if circ_0001766 production depends on QKI (Tables S1 and 2). RIP assays revealed that QKI indeed bound to intron 1 and 3 of *PDIA4* (Fig. 7A). We then knock down QKI using si-QKI in HCT116 cells, verified by western blot (Fig. 7B). Silencing QKI significantly impaired the expression of circ_0001766 and *PPP1R3C*, without affecting *PDIA4* expression, while increasing miR-1203 expression (Fig. 7C). Under hypoxia treatment, QKI protein levels were downregulated, consistent with the downregulated mRNA expression of *QKI* and *PPP1R3C*, as well as circ_0001766 (Fig. 7D, E). Then, we also verified that *QKI* showed markedly downregulated expression in CRC tissues compared with adjacent tissues (Fig. 7F). IHC further validated the low protein expression of QKI in CRC tissues (Fig. 7G). And *QKI* showed a positive correlation with circ_0001766 and *PPP1R3C* expression in CRC, while a negative correlation with miR-1203 (Fig. 7H). Importantly, low *QKI* levels were significantly associated with shortened OS and PFS time in CRC patients (Fig. 7I). Taken together, these data elucidated that QKI binds to introns 1 and 3 of *PDIA4* to promote circ_0001766 biogenesis. Hypoxia reduces QKI levels leading to the downregulation of circ_0001766 in CRC.

## Discussion

**Fig. 8:**
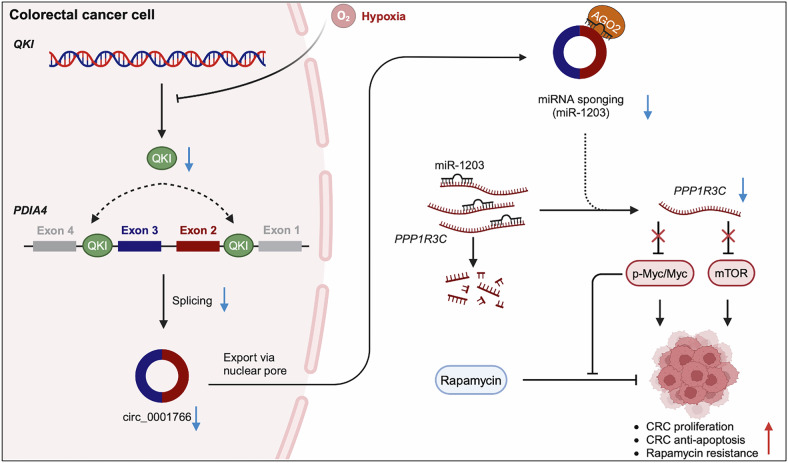
The schematic diagram illustrates that QKI-induced circ_0001766 inhibits colorectal cancer progression and rapamycin resistance via the miR-1203/PPP1R3C/mTOR/Myc axis. Hypoxia induces the downregulation of QKI, which is essential for the biogenesis of circ_0001766 in colorectal cancer cells. QKI increases the formation of circ_0001766 by bringing the circularized exons into proximity. Circ_0001766 acts as a ceRNA, binding to miR-1203 to prevent the degradation of *PPP1R3C* mRNA. Elevated PPP1R3C expression enhances the therapeutic efficacy of rapamycin by inhibiting mTOR signaling and Myc phosphorylation. As a result, circ_0001766 inhibits the proliferation, migration, invasion, and rapamycin resistance of colorectal cancer cells through the miR-1203/PPP1R3C/mTOR/Myc axis.

CircRNAs have exceptional promise as well-established players and diagnostic, prognostic and predictive biomarkers in cancer biology and clinical practice [10, 47]. Here, we revealed a promising role of circ_0001766 in inhibiting CRC progression and overcoming rapamycin resistance. Hypoxia, a hallmark of cancer, inhibited QKI expression which controlled the biogenesis of circ_0001766 via back-splicing of *PDIA4* exons 2 and 3 in CRC. The downregulation of circ_0001766 under hypoxic conditions failed to function as a sponge for miR-1203, leading to the degradation of *PPP1R3C* mRNA. Loss of PPP1R3C resulted in the induction of mTOR and Myc phosphorylation in response to mTOR inhibitor rapamycin, promoting CRC proliferation, migration, invasion and rapamycin resistance (Fig. 8). Restoration of circ_0001766 and PPP1R3C effectively inhibited CRC proliferation, migration and invasion. Additionally, it abrogated rapamycin-induced Myc phosphorylation, thereby resensitizing CRC cells to rapamycin. These results highlight the therapeutic potential of targeting the circ_0001766/miR-1203/PPP1R3C axis to overcome drug resistance and inhibit CRC progression.

Hypoxia is a prevalent and critical feature of most solid tumors, profoundly impacting the biological behavior and malignant phenotype of cancer cells [3, 23]. Hypoxia-responsive circRNAs have been identified in endothelial cells [48], gastric cancer [49], lung adenocarcinoma (LUAD) [50], and cancer cell lines derived from cervical, breast, and lung cancers [51]. In CRC, hypoxia-responsive circ-Erbin has been shown to facilitate CRC aggression and metastasis by enhancing the cap-independent protein translation of HIF-1α [52]. Similarly, circ-133, derived from hypoxic CRC exosomes, promotes CRC metastasis via the miR-133a/GEF-H1/RhoA axis and has been correlated with disease progression, underscoring its potential as a biomarker [53]. While most hypoxia-responsive circRNAs are upregulated in cancers, the mechanisms and roles of downregulated circRNAs remain less understood [54]. Our study demonstrated that hypoxia induces the downregulation of circ_0001766 in CRC cells, promoting CRC proliferation, migration, invasion, and resistance to rapamycin. These findings highlight the diverse roles of hypoxia-responsive circRNAs in cancer biology. QKI, an RNA-binding protein, plays a pivotal role in circRNA biogenesis by binding to specific QKI-binding motifs within RNA molecules [7]. It has been found that hypoxia can downregulate QKI expression by inducing miR-5100 in head and neck squamous cell carcinoma [55]. Our study extends this understanding to CRC, demonstrating that hypoxia microenvironment also leads to the downregulation of QKI in CRC cells, which correlates positively with reduced expression of circ_0001766. Previous studies have shown that QKI binds upstream and downstream of SMARCA5, ZEB1 and NDUFB2 RNA to promote circRNA formation in lung cancer [56], prostate cancer [57], and hepatocellular carcinoma [58]. More interestingly, QKI has been found to bind to the introns 2 and 4 of *PDIA4* mRNA resulting in a novel circRNA generation in gastric cancer [46]. In our study, we found that QKI binds to the introns 1 and 3 of *PDIA4* mRNA, leading to the formation of circ_0001766 in CRC. Therefore, the low expression of circ_0001766 is due to the diminished levels of QKI under hypoxia CRC microenvironment.

CircRNAs located in the cytoplasm can participate in post-transcriptional gene regulation through sponging of miRNAs, thereby preventing specific miRNAs from interacting with and repressing their target mRNAs [37]. The covalent circular structure of circ_0001766 has been validated in oral squamous cell carcinoma, where it has been shown to interact with miR-877-3p, promoting cell proliferation [59]. This aligns with our result that circ_0001766 is predominantly located in the cytoplasm of CRC cells, functioning as a miRNA sponge. Our integrative analysis using the circBank and circInteractome databases predicted miR-1203 and miR-892a as potential circ_0001766-targeted miRNAs. In contrast, the previous study identified miR-877-3p using TargetScan and miRanda, though the interaction between circ_0001766 and miR-877-3p was not experimentally validated [59]. Given the controversies surrounding miRNA sponging, we conducted a series of experiments to confirm the interaction between circ_0001766 and miR-1203. Using RIP, RNA-FISH, dual-luciferase assays, and functional biological experiments, consistent with established standards in circRNA research [37], we solidly demonstrated that circ_0001766 indeed sponges miR-1203 in CRC cells. The pro-oncogenic role of miR-1203 in CRC is further supported by its involvement in hepatocellular carcinoma metastasis and its association with poor prognosis [60]. While these findings highlight the functional significance of circ_0001766 in CRC through its regulation of miR-1203, it is noteworthy that the use of miR-1203 inhibitors or mimics does not fully restore the associated phenotypes. This suggests the possibility of additional interactions, including a potential regulatory relationship between circ_0001766 and miR-892a, as predicted by our analysis using circBank and circInteractome databases. Indeed, a circRNA can exert tumor-suppressive or oncogenic functions by acting as a miRNA sponge for multiple distinct miRNAs, rather than solely containing multiple binding sites for a single miRNA. For example, the oncogenic circCCDC66 contains binding sites for several miRNAs that target oncogenes, including miR-33b and miR-93, which target the MYC oncogene [61]. MiR-892a has been reported to be upregulated in both CRC tissues and cell lines, where it promotes CRC cell proliferation [36]. This raises the possibility that circ_0001766 may act as a sponge for both miR-1203 and miR-892a in CRC. However, further experimental validation is required to confirm this dual regulatory role and its implications for CRC progression.

PP1 and PP2A, the most abundant serine-threonine phosphatases, are families comprising hundreds of protein serine/threonine phosphatases, assembled from a few catalytic subunits in combination with a highly diverse array of regulators [42]. Through integrative analysis for miR-1203 target genes, we identified *PPP1R3C* as the target gene of miR-1203. PPP1R3C encodes a carbohydrate-binding protein that is a subunit of the PP1 complex, which catalyzes reversible protein phosphorylation [42]. Differences in the activities and properties of various PPP1R3 proteins, resulting from somatic mutations and genetic polymorphisms in the PPP1R3 gene, have been implicated in human carcinogenesis and disease susceptibility [62]. Notably, PPP1R3C methylation has been detected at high levels in CRC plasma samples and is particularly useful for early-stage CRC detection [63]. As a hypermethylated gene in CRC, PPP1R3C is thought to play a critical role in cancer cell growth, potentially in association with glucose levels [64]. Our findings also revealed that PPP1R3C is downregulated in CRC and involved in the inhibition of CRC progression. However, the mechanisms by which PPP1R3C contributes to CRC progression and drug resistance remain largely unknown. Another most abundant serine/threonine phosphatases, PP2A, has been reported to show a promising utility, for example, combining PP2A activation with mTOR inhibition reduces c-Myc expression, thereby inhibiting proliferative signaling and inducing cell death in pancreatic ductal adenocarcinoma [65]. In CRC, restoring hypermethylated PPP2R2B, which encodes PP2A, abrogates rapamycin-induced Myc phosphorylation, thus resensitizing cells to rapamycin [28]. Here, we found that overexpression of PPP1R3C inhibited mTOR levels and Myc phosphorylation, suggesting a critical role of protein phosphatase family in rapamycin related pathways. Rapamycin treatment in CRC increased Myc/p-Myc levels, whereas overexpression of circ_0001766 and PPP1R3C with rapamycin treatment successfully inhibited Myc/p-Myc, leading to a significant reduction in CRC proliferation and anti-apoptosis. This presents a combinatorial strategy targeting circ_0001766 or PPP1R3C alongside mTOR inhibition in CRC.

More interestingly, another potential target by circ_0001766, miR-892a, has been revealed to reduce PPP2R2A expression and promote CRC proliferation [36]. From Targetscan database, potential target genes of miR-892a include PPP2R2B. Therefore, circ_0001766 likely serves as a sponge for both miR-1203 and miR-892a, affecting the serine/threonine phosphatase family, including PPP1R3C, PPP2R2A, and PPP2R2B. More evidence is needed to establish the direct interaction between circ_0001766, miR-1203/miR-892a, and the downstream serine/threonine phosphatase family members. Future research should focus on further validating these interactions and exploring the therapeutic application of circ_0001766 modulation in clinical settings.

## Conclusion

In summary, we identified a critical network with circ_0001766 as the central molecule in CRC, which is suppressed due to hypoxia-induced downregulation of QKI in CRC. The loss of circ_0001766 failed to sponge miR-1203, leading to downregulation of PPP1R3C, which in turn prevented the inhibition of mTOR and Myc phosphorylation. The therapeutic potential of targeting circ_0001766 or PPP1R3C is underscored by the ability to restore sensitivity to rapamycin and inhibit key oncogenic pathways, such as mTOR and Myc phosphorylation. Overall, our findings markedly contribute to understanding the molecular mechanisms underlying CRC progression and resistance to rapamycin, opening new avenues for therapeutic targets for CRC and understanding the mechanisms of rapamycin resistance.

## Methods

### Clinical specimens

Paired colorectal cancer (CRC) tissues and adjacent non-tumorous tissues (ANT) were collected from 65 CRC patients who underwent surgery at Xiangya Hospital, Central South University, Changsha, China. A sample size of 65 pairs was chosen based on preliminary statistical power calculations, ensuring adequate power (80%) to detect differences with a significance level of 0.05. Inclusion criteria included patients with histologically confirmed CRC who underwent surgery. Exclusion criteria included patients with a history of other cancers or those who received neoadjuvant chemotherapy or radiotherapy. Ethical approval was obtained from the Ethics Committee of Xiangya Hospital (Approval ID: 202103218). All CRC diagnoses were confirmed through histological and pathological analysis. Tumor staging was determined according to the latest version of the tumor-node-metastasis (TNM) system. Detailed clinical characteristics are provided in Table 1. The samples were rapidly frozen in liquid nitrogen and stored at −80 °C for subsequent RT-PCR and microarray [21].

**Table 1:** Clinical characteristics of CRC patients with low and high circ_0001766 expression.

	Group	Cases	Circ_0001766 Low	Circ_0001766 high	*P* value
Age	≤53	26	16	10	0.7988
	>53	39	22	17	
Gender	Male	41	25	16	0.6122
	Female	24	13	11	
T grade	T1 + T2	11	7	4	0.7510
	T3 + T4	54	31	23	
Tumor size	≤5 cm	38	26	12	0.0746
	>5 cm	27	12	15	
Stage	I-II	29	15	14	0.4480
	III-IV	36	23	13	
Distant metastasis	M0	35	17	19	0.0405^*^
	M1	30	21	8	
Lymphatic invasion	Negative (N0)	36	21	15	0.9814
	Positive (N1-N3)	29	17	12	

*Statistically significant difference (*P* < 0.05).

### Cell lines and cell culture

Human colon cancer cell lines (LoVo, Caco2, HCT8, HCT116, SW480, SW620, HT29) and healthy fetal human colon (FHC) cells were procured from the Institutes of Biomedical Sciences (IBS, Shanghai, China) and handled according to the supplier’s recommendations. LoVo, Caco2, and FHC cells were cultured in MEM Medium (Gibco, USA), while SW480 and SW620 were maintained in L-15 Medium (Gibco, USA). HCT116 and HCT8 were cultured in RPMI‐1640 Medium (Gibco, USA). HT29 was cultured in McCoy’s 5A Medium (Gibco, USA). The culture media were supplemented with 10% fetal bovine serum (Gibco, USA) and 1% penicillin and streptomycin (Gibco, USA). All cell lines were maintained at 37 °C in a humidified atmosphere containing 5% CO_2_. Cell lines were authenticated via STR profiling and routinely tested for mycoplasma contamination.

### RNA isolation and quantitative real-time reverse transcription polymerase chain reaction (RT-PCR)

Total RNA was isolated using TRIzol Reagent (Cat# 15596018CN, Invitrogen, USA), and cDNA amplification (1 µg per sample) was carried out using the Prime Script Kit (Cat# AG11705, Accurate Biology, China) as per the manufacturer’s instructions. Real-time PCR was conducted in triplicate using a SYBR Green fluorescent-based assay (Cat# AG11701, Accurate Biology, China) on a ViiATM7 RT-PCR system (Applied Biosystems, Carlsbad, CA). The primers for real-time PCR were detailed in Table S3. The relative mRNA expression levels were determined using the 2 − (ΔΔCt) method and normalized to the internal control (GAPDH), where ΔCt = Ct(circRNA)–Ct(U6) or ΔCt = Ct (target genes)–Ct (GAPDH) and ΔΔCt = ΔCt(case) -ΔCt(control). The primers for real-time PCR were listed in Supplementary information.

### Sanger sequencing and RNase R treatment

The amplification products of circ_0001766 were validated through Sanger sequencing provided by Geneseed Biotech (Guangzhou, China). RNase R (Geneseed Biotech, Guangzhou, China) was utilized to degrade linear RNA. RNA extracted from Caco2 and HCT116 cells was divided into two groups: one group was subjected to treatment with RNase R, while the other group served as the control. Both groups were then exposed to 2.5U/μg RNase R at 37 °C for 30 min. Subsequently, circ PDIA4 and linear PDIA4 were analyzed using reverse transcription and qRT‐PCR, with the control group utilizing GAPDH as an internal reference. The entire process was independently repeated three times in triplicate.

### Lentiviral transduction

A lentiviral vector, pHBLV-CMV-MCS-EF1-Zsgreen1-T2A-puro, containing human circ_0001766 was constructed by Hanbio Biotechnology (Shanghai, China). Short hairpin RNA (shRNA) targeting circ_0001766 or a random sequence was synthesized and inserted into the lentiviral vector pHBLV-U6-MCS-CMV-ZsGreen-PGK-PURO, also from Hanbio Biotechnology (Table S4). CRC cells were then transduced with the appropriate lentiviruses. Stable transduced cells were selected using puromycin (2 μg/mL) for 2 weeks. The expression level of circ_0001766 was determined by RT-qPCR.

### Plasmid construction and transfection

Small interfering RNA (siRNA) duplexes targeting circ_0001766, PPP1R3C, RBM20, MBNL1, and QKI were designed and synthesized by Hanbio Biotechnology (Shanghai, China) (Table S4). A nonhomologous negative control RNA duplex (NC) for siRNAs was obtained from Hanbio Biotechnology. The miRNA mimic and inhibitor were purchased from RiboBio Biotech (Guangzhou, China). CRC cells were transfected with the specified plasmids and siRNAs using Lipofectamine 3000 reagent (Invitrogen, Carlsbad, USA), following the manufacturer’s protocol.

### Western blot

Protein lysates from cultured cells were extracted in lysis buffer (P0013B, Beyotime, Shanghai, China), and their concentrations were determined using the BCA Protein Assay Kit (Beyotime Biotechnology, Shanghai, China). Equal amounts of denatured protein lysates were subjected to SDS-PAGE electrophoresis, transferred onto PVDF membranes (Millipore, IPFL00010), and blocked in 5% bovine serum albumin at room temperature for 1 h. Subsequently, the membranes were incubated with primary antibodies against anti-GAPDH (Cat#60004-1-Ig, Proteintech, China), anti-PPP1R3C (Cat# ab251771, Abcam, USA), and anti-QKI (Cat# ab126742, Abcam) at room temperature for 1 h, followed by washing three times for 10 min each with 0.1% TBS Tween 20 (0.1 M TBS: 3 g Tris, 0.2 g KCl, 8 g NaCl, pH 7.4). The PVDF membranes were then incubated with the respective HRP-conjugated secondary antibodies, goat anti-mouse IgG (Cat# ab6728, Abcam, USA) or goat anti-rabbit IgG (Cat#SA00001-2, Proteintech, China), at room temperature for 1 h. Protein bands were visualized using the ChemiDocXRS+ system. The uncropped western blots are included in the supplemental material.

### Histology and immunohistochemistry (IHC) analysis

Tumor tissues were fixed in 4% paraformaldehyde and embedded in paraffin for immunohistochemical (IHC) analysis. Paraffin-embedded xenograft tumors were initially processed at 60 °C for 2 h, followed by dewaxing with 100% xylene and rehydration with varying concentrations of ethanol. Subsequently, the slides were subjected to EDTA treatment at 121 °C for antigen retrieval, and endogenous peroxidase activity was quenched with 3% hydrogen peroxide at room temperature. The sections were then incubated overnight at 4 °C with primary antibodies, followed by incubation with corresponding secondary antibodies at room temperature for 2 h the next day. Macroscopic nodules were photographed for image acquisition.

### Cell viability assay, colony formation assays, and Edu

For the cell viability assay, siRNA- or plasmid-transfected CRC cells (2.0 × 10^3^ cells per well) were seeded into 96-well plates. CCK-8 solution (10 μL) was added to each well and incubated for 2 h following the manufacturer’s instructions (CCK-8 Kit, FUDE, Hangzhou, China). Absorbance was then measured at 450 nm. Each assay was conducted in triplicate. For the colony formation assay, CRC cells transfected with siRNA or plasmid were plated into 6-well plates (1 × 10^3^ cells per well) and cultured for 2 weeks to allow colony formation. The cells were fixed with 4% paraformaldehyde for 15 min and stained with 0.1% crystal violet for 20 min at room temperature. Colonies were manually counted under a microscope. Each assay was performed in triplicate. To assess cell proliferation viability, the EdU assay was conducted using the Cell-Light TM EdU Kit (Cat. No. C10312, RiboBio, China) following the manufacturer’s instructions. Transfected CRC cells were seeded in 12-well plates and incubated with 50 mM EdU for 2 h. Subsequently, the cells were fixed and stained for 30 min. Each assay was performed in triplicate.

### Apoptosis and transwell assays

The Annexin V-FITC Apoptosis Detection Kit I (Cat# 556547, BD Pharmingen, USA) was used to detect cell apoptosis according to the manufacturer’s protocol. Flow cytometry was performed using the FACS Canto II flow cytometer (BD Biosciences) to assess cell apoptosis. In brief, cells resuspended in 1× Binding Buffer were added to tubes containing Annexin V-FITC reagent (5 µl) and PI reagent (5 µl) and incubated in the dark at room temperature for 15 min. Flow cytometry was conducted within 1 h. Following transfection, 5 × 10^4^ CRC cells in 100 μl serum-free culture medium were seeded into the upper chambers of 8 μm pore size Transwells (Cat# 3374, Corning, USA), with 600 μl complete culture medium added to the lower chamber. For invasion assays, 5 × 10^4^ cells were seeded into the Transwell invasion chambers with a Matrigel-coated membrane of 0.8 mm pore size. After 24 h of incubation, cells in the upper chamber were removed with a cotton swab, while those on the lower chamber were fixed in 2% paraformaldehyde for 10 min and stained with crystal violet. Migration or invasion cell counts were performed under a microscope (N2-DMi8, Leica, Germany) in six randomly selected areas.

### Dual-luciferase reporter assay

The binding sites of circ_0001766 with miR-1203, as well as the binding site of miR-1203 with PPP1R3C, were separately cloned into the luciferase reporter vector (OBiO Technology, China). HT29 and HCT116 cells were seeded into 96-well plates and were co-transfected with a mixture of 60 ng of firefly luciferase reporter, 6 ng of pSI-Check2 Renilla luciferase reporter, and miR-1203 mimic or inhibitor. After 48 h of incubation, the firefly and Renilla luciferase activities were measured with the Dual-Luciferase Reporter Assay System (E1910, Promega, USA).

### Xenograft tumor

The subcutaneous xenograft models were handled in accordance with ethical guidelines, and CRC cells transfected with LV-circ_0001766 and sh-circ_0001766 or the control vector were subcutaneously implanted into the right flank regions of female BALB/c nude mice (4–5 weeks old, 6 mice per group, 5 × 10^6^ cells per mouse). Tumor volume was assessed every 3 days using the formula: V (mm^3^) = (L × W^2^) × 0.5 (L: tumor length, W: width). After 3 weeks, the mice were euthanized, and the tumors were excised and quantified. Mice were randomly assigned to experimental groups using computer-generated random numbers. Researchers performing tumor volume measurements were blinded to the group allocation. All animal experimental protocols were approved by the Medical Laboratory Animal Care Committee of Central South University (Approval ID: 202103218). Experiments were conducted following the Institutional Animal Care and Use Committee (IACUC) guidelines.

### Lung metastasis model and bioluminescent in-vivo imaging

BALB/c-nude mice aged 6 weeks received an intravenous injection of 100 μL of CRC cell single-cell suspension (5 × 10^6^/mL) via the tail vein. After 28 days, the mice were euthanized, and lung tissues were harvested to observe lung metastatic foci. On the 28th day following the establishment of the subcutaneous lung metastasis mouse model described above, bioluminescence imaging was conducted to assess tumor growth. Briefly, VivoGloTM luciferin (CAT# P1041, Promega, USA) was intraperitoneally injected into tumor-bearing mice at a dosage of 150 mg/kg or 10 μl/g of body weight. Subsequently, bioluminescent signals were captured using an in-vivo imaging system (FX Pro, USA) within 10–12 min post-injection. Mice were randomly assigned to groups, and the bioluminescence data were analyzed by researchers blinded to group allocation.

### RNA-fluorescence in situ hybridization (FISH)

FISH was performed to assess the subcellular localization of circ_0001766 and miR-1203 in colorectal cancer (CRC) cells and tissues using the FISH Kit (Cat#C10910, RiboBio, China). CRC cells were subjected to hybridization with the Cy3-labeled circ_0001766 probe (RiboBio, Guangzhou, China) and FAM-labeled miR-1203 probe (RiboBio, China), respectively, according to the manufacturer’s instructions. 4′,6-diamidino-2-phenylindole (DAPI) was utilized for nuclear staining, and TCS SP8 confocal microscope (Leica, Wetzlar, Germany) was utilized to observe the sections.

### RNA immunoprecipitation (RIP)

According to the manufacturer’s instructions, the Magna RIP RNA-Binding Protein Immunoprecipitation Kit (Millipore, USA) was utilized. Briefly, cells were cross-linked and lysed, and the lysates were treated with DNase I for 10 min followed by centrifugation at 12,000× *g* for 30 min. Immunoprecipitation of samples was performed using QKI rabbit monoclonal antibody (Cat# ab126742, Abcam, USA) or control goat anti-mouse IgG antibody (Cat# ab6708, Abcam, USA), and Protein G Magnetic Beads (Life Technologies) were added. After bead washing, RNA was eluted using phenol:chloroform for subsequent qRT-PCR analysis, and input was normalized for comparison (fold change calculation).

### Statistical analysis

Statistical analyses were conducted using GraphPad Prism 9.0 software. Quantitative data are presented as mean ± standard error of mean. A two-tailed unpaired or paired Student’s *t* test and ANOVA (Dunnett’s or LSD post hoc test) were employed to assess differences in parameter variables between groups. Pearson correlation analysis was performed to evaluate the correlation among mRNAs, miRNAs, and circRNAs. Overall survival (OS) and progression-free survival (PFS) were determined using the Kaplan–Meier method and assessed with the log-rank test. The Cox proportional hazard regression model was utilized to identify independent prognostic factors for CRC. All statistical tests were two-sided, and *P* < 0.05 was considered statistically significant. All experiments were repeated at least three times independently to ensure reproducibility. Detailed data, including raw values and analysis code, are available upon request.

## Supplementary information

Supplementary Material file 1

Supplementary Material file 2

## Data Availability

The data presented in the study are deposited in the GEO repository, accession number GSE205643. All other data supporting the findings of this study are present in the paper and Supplementary Information.
