# Supplementary material for: QKI-induced circ_0001766 inhibits colorectal cancer progression and rapamycin resistance by miR-1203/PPP1R3C/mTOR/Myc axis
Source: Cell Death Discov. 2025 Apr 23;11:192. doi: 10.1038/s41420-025-02478-w (PMC12015279; doi:10.1038/s41420-025-02478-w)
Supplement: Supplementary file 1 — Supplementary Material file 1 [file 41420_2025_2478_MOESM1_ESM.pdf]

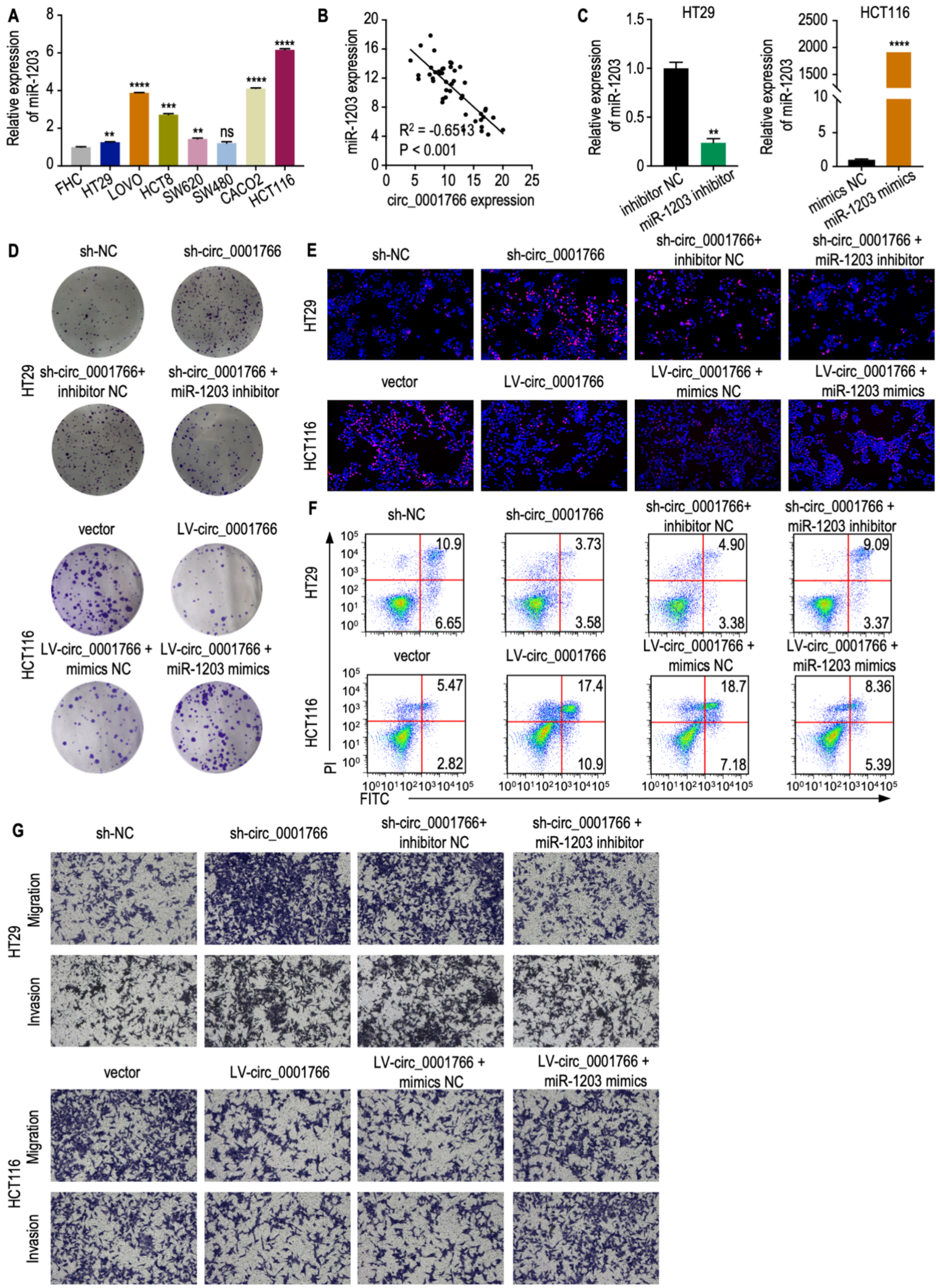

Fig S1. Circ_0001766 functioned as a sponge for miR-1203. A, The relative expression levels

of miR-1203 in normal epithelial cell (FHC) and CRC cell lines. The *p* value was determined by a two-tailed unpaired Student *t* test. **B,** The Pearson correlation analysis between circ_0001766 and miR-1203 expression in CRC tissues. **C,** Relative expression of miR-1203 in HT29 cells with control or miR-1203 inhibitors (left) and HCT116 cells with control and miR-1203 mimics (right). The *p* value was determined by a two-tailed unpaired Student *t* test. **D-E,** Detection of circ_0001766 inhibiting CRC cell proliferation through sponging miR-1203 by colony formation assay (**D**) and Edu assay (**E**). The *p* value was determined by a two-tailed unpaired Student *t* test. **F,** Flow cytometry showing circ_0001766 promoted CRC cell apoptosis through sponging miR-1203. The *p* value was determined by a two-tailed unpaired Student *t* test. **G,** The inhibition ability of circ_0001766 through sponging miR-1203 for CRC cell migration and invasion was shown by transwell assay. The *p* value was determined by a two-tailed unpaired Student *t* test. Data represent mean ± SEM. ns, not significant; **, $P<0.01$; ***, $P<0.001$; ****, $P<0.0001$.

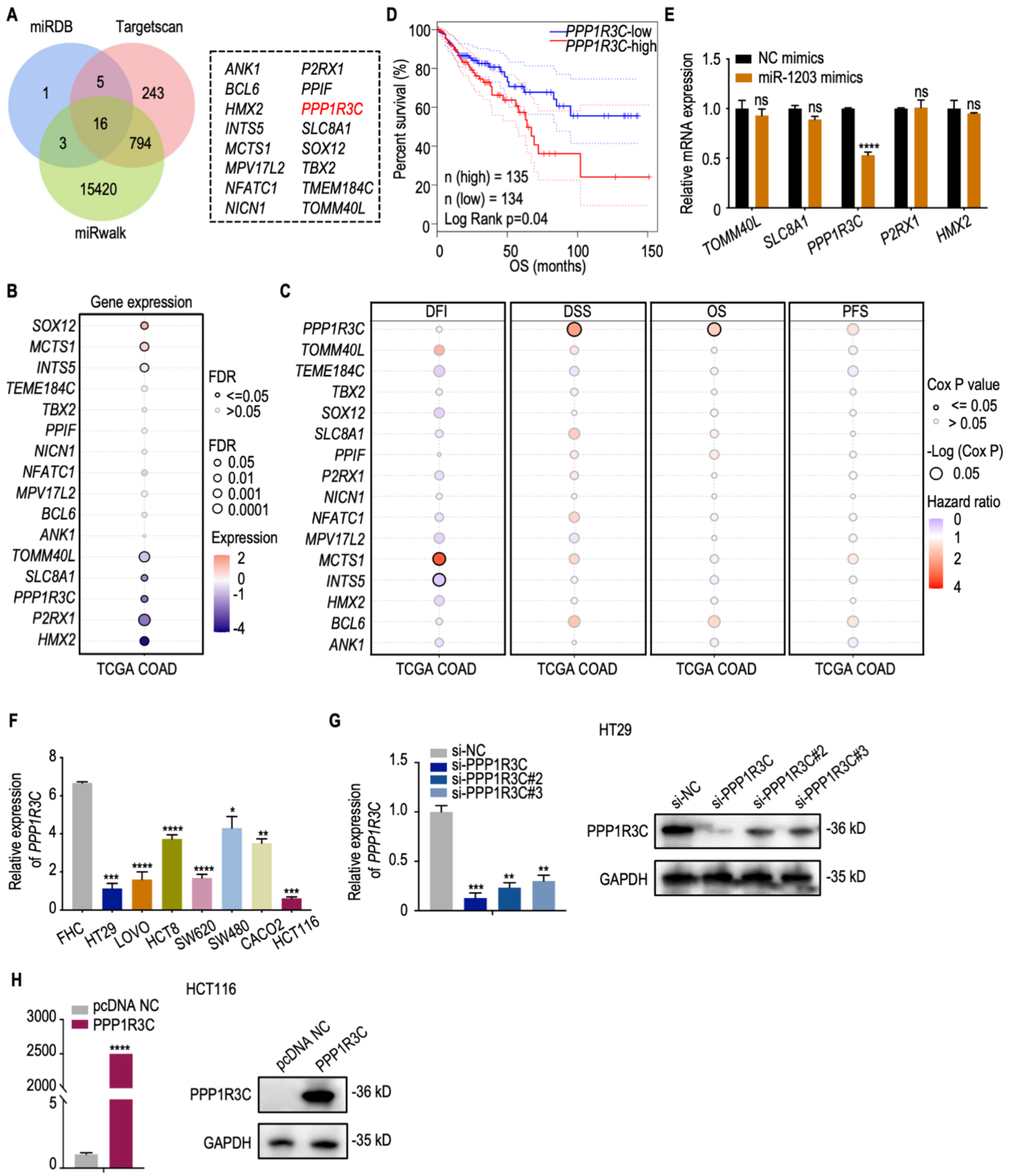

Fig S2. Prediction of miR-1203 targeted genes. A, Venn plot showing prediction of miR-1203 targeted mRNAs in miRDB, Targetscan and miRwalk database. **B,** Gene expression of target genes in (**A**) in TCGA-COAD dataset. The bubble plot presents the FDR through bubble color and size.

The row is the gene symbol and the column is the selected cancer types. The scale bar color from blue to red represents the gene expression. **C,** Disease-Free Interval (DFI), Disease-Specific Survival (DSS), Overall Survival (OS) and Progression-Free Survival (PFS) of target genes in (**A**) in TCGA-COAD dataset. The bubble plot presents the COX P value through bubble color and size. The row is the gene symbol and the column is the selected cancer types. The scale bar color from red to purple represents the hazard ratio. **D,** OS of PPP1R3C in TCGA-COAD dataset stratified by *PPP1R3C* expression level. The log-rank test was used for survival comparison. **E,** Relative expression of select genes in (**A**) in HCT116 cells with control or miR-1203 mimics. The *p* value was determined by a two-tailed unpaired Student *t* test. **F,** The relative expression levels of *PPP1R3C* in normal epithelial cell (FHC) and CRC cell lines. The *p* value was determined by a two-tailed unpaired Student *t* test. **G,** Relative expression of *PPP1R3C* mRNA (left) and PPP1R3C protein (right) in HT29 cells with control or siRNAs (left). The *p* value was determined by a two-tailed unpaired Student *t* test. **H,** Relative expression of *PPP1R3C* mRNA (left) and PPP1R3C protein (right) in HCT116 cells with control and overexpressed circ_0001766 plasmid (right). The *p* value was determined by a two-tailed unpaired Student *t* test. Data represent mean ± SEM. ns, not significant; *, $P<0.05$; **, $P<0.01$; ***, $P<0.001$; ****, $P<0.0001$.

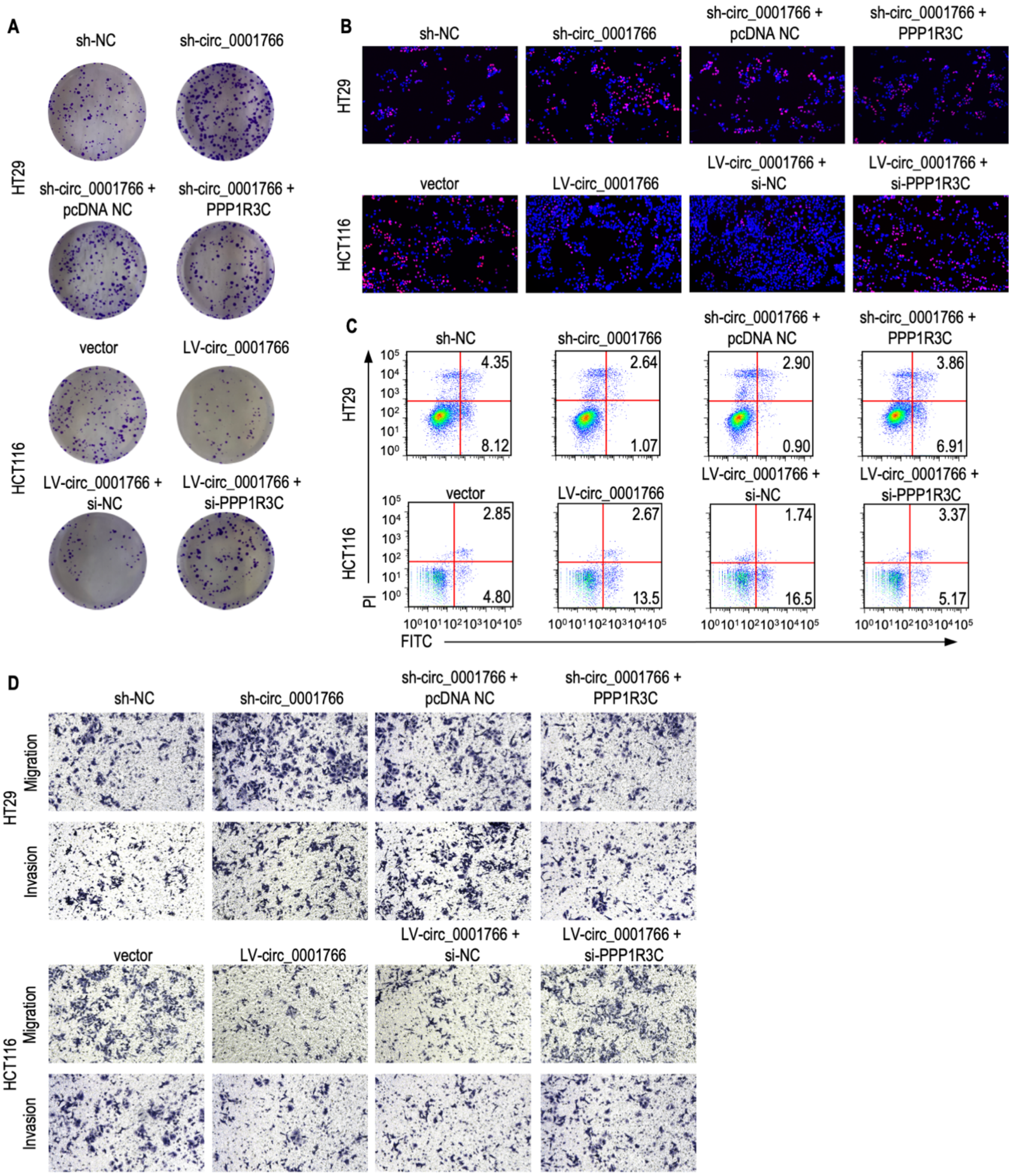

Fig S3. Circ_0001766 functioned through miR-1203/PPP1R3C pathway. A-B, Detection of circ_0001766 inhibiting CRC cell proliferation through increasing expression of *PPP1R3C* by colony formation assay (**A**), and Edu assay (**B**). **C,** Flow cytometry showing circ_0001766

promoted CRC cell apoptosis through PPP1R3C. **D,** The inhibition ability of circ_0001766 through PPP1R3C for CRC cell migration and invasion was shown by transwell assay.

Table S1. Motif analysis and prediction of QKI binding sites in *PDIA4* intron 1.

Position	Genomic	Motif	Occurrence	Z-score	P-value
321	chr7:149028000	acuaay	uuuugcagcuggguccuucgccuua**auuuac**agauuu ucgucagguccaggcuaac	2.022	2.16e-02
346	chr7:149027975	acuaay	auuuacagauuuucgucagguccag**gcuaac**uccaggc gaacgcgcccaggcagug	2.043	2.05e-02
665	chr7:149027656	acuaay	augaagcgcguacuccaguaggaau**aguaac**aacuacu ucaugguccccugcauuuu	2.707	3.39e-03
668	chr7:149027653	acuaay	aagcgcguacuccaguaggaauagu**aacaac**uacuucau gguccccugcauuuuucu	1.859	3.15e-02
672	chr7:149027649	acuaay	gcguacuccaguaggaauaguaaca**acuacu**ucauggu cccugcauuuuucuucga	1.804	3.56e-02
723	chr7:149027598	acuaay	uucgaagugagcuagcugcagacau**aagaac**ucugaag auuaggaagugcuaauua	1.685	4.60e-02
746	chr7:149027575	acuaay	auaagaacucugaagauuaggaagu**gcuaau**uaaggaa aaguuguuauuccuagag	2.011	2.22e-02
750	chr7:149027571	acuaay	gaacucugaagauuaggaagugcua**auuaag**gaaaagu uguuauuccuagagaaua	1.837	3.31e-02
830	chr7:149027491	acuaay	cuucuuccucucugcccuuacuuuu**aaucac**uagcuuu uuaaccuucugaguuugu	2.380	8.66e-03
834	chr7:149027487	acuaay	uuccucucugcccuuacuuuuaauc**acuagc**uuuuuaa ccuucugaguuuguucua	2.739	3.08e-03
842	chr7:149027479	acuaay	ugcccuuacuuuuaaucacuagcuu**uuuaac**cuucug aguuuguucuaauauaaau	2.859	2.12e-03
938	chr7:149027383	acuaay	acuuuauacucuuucaaacagauug**aguaac**uucugcg ugggucacugauucaaaa	2.783	2.69e-03
957	chr7:149027364	acuaay	agauugaguaacuucugcgugggguc**acugau**ucaaaaa gagucuuugcccaaauag	2.098	1.80e-02
995	chr7:149027326	acuaay	gagucuuugcccaaauagaaaagga**aguaaa**aacccac acucggcgagaaaaacac	1.967	2.46e-02
998	chr7:149027323	acuaay	ucuuugcccaaauagaaaaggaagu**aaaaac**ccacacu cggcgagaaaaacaccug	1.891	2.93e-02
1018	chr7:149027303	acuaay	gaaguaaaaacccacacucggcgag**aaaaac**accugccg aaaggaauccgauagcg	1.891	2.93e-02
2119	chr7:149026202	acuaay	ucaucccugagggcaucccucggcc**auucac**ugucaaa accugucauagauucacu	1.913	2.79e-02
2143	chr7:149026178	acuaay	cauucacugucaaaaccugucauag**auucac**ugauaac uuguccaacagauuuauu	2.783	2.69e-03
2147	chr7:149026174	acuaay	cacugucaaaaccugucauagauuc**acugau**aacuugu ccaacagauuuauuucaa	2.239	1.26e-02
2150	chr7:149026171	acuaay	ugucaaaaccugucauagauucacu**gauaac**uuguccа acagauuuauuucaaguc	2.207	1.37e-02
2184	chr7:149026137	acuaay	uccaacagauuuauuucaagucugc**acuacc**ucacccc ucaaguguuucaagucuc	2.130	1.66e-02
2312	chr7:149026009	acuaay	augcccuccuucccccaacaugguu**acuaaa**uacaccc uaagcucugaugguuuaa	1.880	3.01e-02
2480	chr7:149025841	acuaay	ugguauugguuuguuuagauuuaaa**aguaag**uagaaa uucaccuauuuuucgugcu	2.076	1.89e-02
2491	chr7:149025830	acuaay	uguuuagauuuaaaaguaaguagaa**auucac**cuauuu uucgugcuaaggaacuguu	2.587	4.84e-03
2512	chr7:149025809	acuaay	agaaauucaccuauuuuucgugcua**aggaac**uguuaaa cucuccuccucuuuucau	2.141	1.61e-02
2825	chr7:149025496	acuaay	gcccagcacagugccugguacauac**aauaaa**uaccagc uaucaugguuaccguagc	2.207	1.37e-02
2829	chr7:149025492	acuaay	agcacagugccugguacauacaaua**aauacc**agcuauc augguuaccguagcugau	2.130	1.66e-02
4183	chr7:149024138	acuaay	gcaauucuccugccucagccuccca**aguagc**uaagauu acaggcaugcaccaacau	2.022	2.16e-02
4206	chr7:149024115	acuaay	caaguagcuaagauuacaggcaugc**accaac**augccca gcuaauuuuuguacuuuu	2.522	5.83e-03
4658	chr7:149023663	acuaay	cugugauuccuaccuggaagaguug**auuaag**uucccc	1.772	3.82e-02

			agugcauuauuuccgaccu		
4785	chr7:149023536	acuaay	gaguggauauggauggucccaaagcacuacuuaacugc cagccggggccccuggug	1.870	3.07e-02
4789	chr7:149023532	acuaay	ggauauggauggucccaaagcacuacuuaacugccagc cggggccccugguggucu	2.109	1.75e-02
4877	chr7:149023444	acuaay	ugucaaaaaucucgauuacuuuugcaccaacccuaaua cuugggcuuagucuuaau	2.261	1.19e-02
4883	chr7:149023438	acuaay	aaaucucgauuacuuuugcaccaacccuaauacuuggg cuuagucuuaaugacauc	2.217	1.33e-02
5712	chr7:149022609	acuaay	ccugaccucauccacagcccaaaacaguaacuagcagcg uucauuuaccuccugga	3.076	1.05e-03
5716	chr7:149022605	acuaay	accucauccacagcccaaaacaguaacuagcagcguuca uuuaccuccuggaaaac	2.500	6.21e-03
5729	chr7:149022592	acuaay	cccaaaacaguaacuagcagcguucauuuaccuccugg aaaacacaucuccagaaa	2.402	8.15e-03
5846	chr7:149022475	acuaay	gcccaguucaguuuacuggugugacaguagccagagaa ccugguuaauaaaaaugg	1.848	3.23e-02
5866	chr7:149022455	acuaay	gugacaguagccagagaaccugguuaauaaaaauggca auuccauguucuuuguau	1.924	2.72e-02
5915	chr7:149022406	acuaay	uuuguaucugugguuuggcaaaaaaaauaaaaauaaaa uaaguugaaccuugaaaau	2.283	1.12e-02
5920	chr7:149022401	acuaay	aucugugguuuggcaaaaaaaaauaaaauaaaaauaaguu gaaccuugaaaauaaaau	2.489	6.41e-03
5925	chr7:149022396	acuaay	ugguuuggcaaaaaaaaauaaaauaaaauaaguugaacc uugaaaauaaaauauaca	2.489	6.41e-03
5943	chr7:149022378	acuaay	aaaauaaaauaaguugaaccuugaaaauaaaauauaca caauuucucacauucuag	2.380	8.66e-03
6054	chr7:149022267	acuaay	caauuucuauauuuguuauccuggaaauuacucaggg gugccuuuauaaguaaaau	1.859	3.15e-02
6077	chr7:149022244	acuaay	gaaauuacucaggggugccuuuauaaguaaaaugaga uuucccgcccucuuccccu	1.935	2.65e-02
6356	chr7:149021965	acuaay	aggaggaggcggcacaucagugagcaauaacguugaac acagacaagcuagggcga	2.043	2.05e-02
6690	chr7:149021631	acuaay	ugaccucccucugaaugaaauuuuaaguagcaaacuua gaacagcuggcaugaggg	1.804	3.56e-02
7130	chr7:149021191	acuaay	gcccggccaggaaaguuuuaaggagagucaccaccagc uuuguaaccagucugggu	1.913	2.79e-02
7145	chr7:149021176	acuaay	uuuuaaggagagucaccaccagcuuuguaaccagucug gguguuuuccacucag	2.043	2.05e-02

Table S2. Motif analysis and prediction of QKI binding sites in *PDIA4* intron 3.

Position	Genomic	Motif	Occurrence	Z-score	P-value
94	chr7:149018898	acuaay	ggaagaaaagcugaggguaccagggacugacuuucucaggaa ccuuagcccucugg	1.848	3.23e-02
353	chr7:149018639	acuaay	aggucaggaguucaagaccagccugaccaaccauggugaaaccc caucucuacuaaa	2.565	5.16e-03
378	chr7:149018614	acuaay	accaacauggugaaaccccaucucuacuaaaaauacaacaauu agcuggguguagu	2.457	7.01e-03
547	chr7:149018445	acuaay	gacagaaugagacucuguuucaaaaaauaaauaaacaggagu guaaggaaaugaca	2.457	7.01e-03
551	chr7:149018441	acuaay	gaaugagacucuguuucaaaaaauaaauaaacaggaguguaa ggaaaugacaucag	2.457	7.01e-03
552	chr7:149018440	acuaay	aaugagacucuguuucaaaaaauaaauaaacaggaguguaag gaaaugacaucaga	2.576	5.00e-03
571	chr7:149018421	acuaay	aaauaaauaaacaggaguguaaggaaaugacaucagagguca cgggucuugguggu	2.315	1.03e-02
1045	chr7:149017947	acuaay	caggcgugaaccaccacgcccgcccaauaauuugcauuucuu acaagcaugcaggg	1.826	3.39e-02
1224	chr7:149017768	acuaay	ucacauuagacacuacaugcauuuuauuaaaugaaugaacaa auaaauuauuugau	3.022	1.26e-03
1233	chr7:149017759	acuaay	acacuacaugcauuuuauuaaaugaaugaacaaauaaauuau uugauuuuuauuua	2.935	1.67e-03
1237	chr7:149017755	acuaay	uacaugcauuuuauuaaaugaaugaacaaauaaauuauuug auuuuuauuuaauuu	2.565	5.16e-03
1240	chr7:149017752	acuaay	augcauuuuauuaaaugaaugaacaaauaaauuauuugauu uuuauuuaauuuuau	2.772	2.79e-03
1700	chr7:149017292	acuaay	gccacugugcccggccucuuauuuuaauaaaauuauuuaau uuuauugaaugaaca	2.511	6.02e-03
1724	chr7:149017268	acuaay	uaauaaaauuauuuaauuuuauugaaugaacaugguauccu uuagcaagggcaaau	2.587	4.84e-03
2009	chr7:149016983	acuaay	cuccucugcuccuuuaagcaauggcacucacuguggcguaua caaagagaauggau	1.739	4.10e-02
2091	chr7:149016901	acuaay	aguuuguugacuagguaauacauuuauaaacuugguaauuc agagagguagauuaa	2.043	2.05e-02
2117	chr7:149016875	acuaay	uaaacuugguaauucagagagguagauuaaugaauagucuu ucuucccugaaccug	1.935	2.65e-02
2272	chr7:149016720	acuaay	aggucaggagaucgagaccauccuggcuaacaugguuaaacc ccaucucuacuaaa	2.576	5.00e-03
2297	chr7:149016695	acuaay	gcuaacaugguuaaaccccaucucuacuaaaaauauaaaaaa uuagcuggaugugg	3.043	1.17e-03
2313	chr7:149016679	acuaay	cccaucucuacuaaaaauauaaaaaauuagcuggauguggug gcagguggcuggug	2.065	1.95e-02
2588	chr7:149016404	acuaay	uuacaguuauuaguauaugucaaauacuauccuguaccuuc auguuuuuauuuaac	2.478	6.61e-03
2613	chr7:149016379	acuaay	acuauccuguaccuucauguuuuuauuuaacagauaugucu uagccauauccauac	1.989	2.34e-02
2647	chr7:149016345	acuaay	uaugucuuagccauauccauacauaaagaacuuaaucuaaaa caaauugaaaguug	2.087	1.84e-02
2663	chr7:149016329	acuaay	ccauacauaaagaacuuaaucuaaaacaaauugaaaguugag uacuucuuuauuuu	2.043	2.05e-02
3527	chr7:149015465	acuaay	auacaucuuaugacguauguaauauaguaacauuagaugug aaugacuaaauaugu	3.054	1.13e-03
3543	chr7:149015449	acuaay	auguaauauaguaacauuagaugugaaugacuaaauaugua uauauuuuguaugug	2.435	7.45e-03
3547	chr7:149015445	acuaay	aauauaguaacauuagaugugaaugacuaaauauguauaua uuuuguauguguuca	2.946	1.61e-03

Table S3. Primer sequences for RT-qPCR.

Gene symbol	Primer Sequence
PDIA4	Forward 5'- TGCCGCTAACAACCTGAGAG -3'
	Reverse 5'- GACGTCCATCATGTGGCTCC -3'
Circ_0001766	Forward 5'- CTCCAGAACCCAGGAAGATT -3'
	Reverse 5'- GCATCATTTAGGACCAAGAC -3'
PPP1R3C	Forward 5'- TTCGAATTTGTGCAGGCAGC -3'
	Reverse 5'- TGAATGTGCCAAGCAAAGCC -3'
TOMM40L	Forward 5'- CAGGCTAACCTCGGCTCTTC -3'
	Reverse 5'- TGACAACGAGCTTCACTCCC -3'
SLC8A1	Forward 5'- TGCTTTGTGCTTCCCACAGA -3'
	Reverse 5'- TCCGTTCCTCCACGCTAGTA -3'
P2RX1	Forward 5'- GTCATCGGGTGGGTGTTTCT -3'
	Reverse 5'- GGGAAGACGTAGTCAGCCAC -3'
HMX2	Forward 5'- AAGCAACCTCTCGGCCTTAC -3'
	Reverse 5'- TTACAGTACAGTACGCGCCC -3'
QKI-RIP-qPCR-a	Forward 5'- TGGCTTGGTTTGCACCTTTG -3'
	Reverse 5'- TCTGCAGCTAGCTCACTTCG -3'
QKI- RIP-qPCR-b	Forward 5'- ACCTTCTTCCTCTCTGCCCTT -3'
	Reverse 5'- TGACCCACGCAGAAGTTACTC -3'
QKI- RIP-qPCR-c	Forward 5'- TTCTTGAGAGGCCTGGGAGA -3'
	Reverse 5'- GCTCACCCTAGGAGCAACTG -3'
QKI- RIP-qPCR-d	Forward 5'- TGATGGTTTAAGCCTCCGGT -3'
	Reverse 5'- AGCAGGCAACAAACAGGATCA -3'
QKI	Forward 5'- GCAAAATAGAGGCAAGCCCA -3'
	Reverse 5'- CCTTCTGCTGCAGGTACCAA -3'
U6	Forward 5'- CTCGCTTCGGCAGCACA -3'
	Reverse 5'- AACGCTTCACGAATTTGCGT -3'
GAPDH	Forward 5'- CGGAGTCAACGGATTTGGTCGTAT -3'
	Reverse 5'- AGCCTTCTCCATGGTGGTGAAGAC-3'

Table S4. Sequences of shRNAs and siRNAs.

Name	Primer Sequence (5’-3’)
circ_0001766 shRNA#1	GATCCGAGAACCCAGGAAGATTCTTCTTTCAAGAGAAGAAGAATCTTCCTGGGTTCTTTTTTTG
circ_0001766 shRNA#2	GATCCGGAACCCAGGAAGATTCTTCTATTCAAGAGATAGAAGAATCTTCCTGGGTTCTTTTTTG
circ_0001766 shRNA#3	GATCCGGGAAGATTCTTCTAACAGAGATTCAAGAGATCTCTGTTAGAAGAATCTTCCTTTTTTG
sh NC	GATCCGTTCTCCGAACGTGTCACGTAATTCAAGAGATTACGTGACACGTTCGGAGAATTTTTTC
PPP1R3C siRNA#1	AAGUGAUACGGAUCUGAACTT
PPP1R3C siRNA#2	AUUUAUUCACAAAAUGUCGTT
PPP1R3C siRNA#3	UUAAUUCAUCGAUAAGAGGTT
QKI siRNA	UAACUGAACAAUAGGUCCCTT
si NC	ACGUGACACGUUCGGAGAATT
