# Supplementary material for: QKI-induced circ_0001766 inhibits colorectal cancer progression and rapamycin resistance by miR-1203/PPP1R3C/mTOR/Myc axis
Source: Cell Death Discov. 2025 Apr 23;11:192. doi: 10.1038/s41420-025-02478-w (PMC12015279; doi:10.1038/s41420-025-02478-w)

File S2

Uncropped blots for Figure 5F

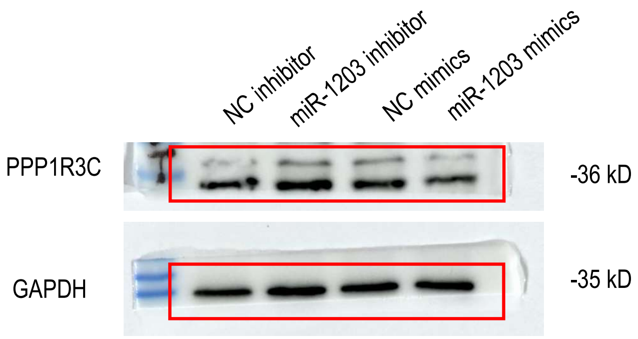

File S2

Uncropped blots for Figure 6A

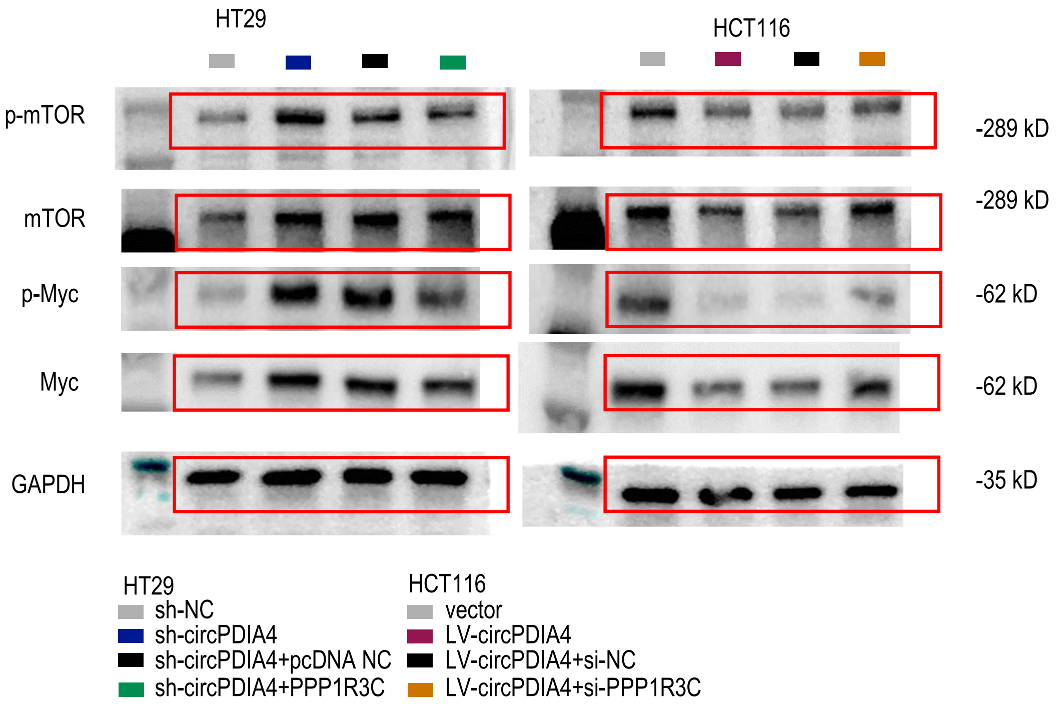

Uncropped blots for Figure 6B

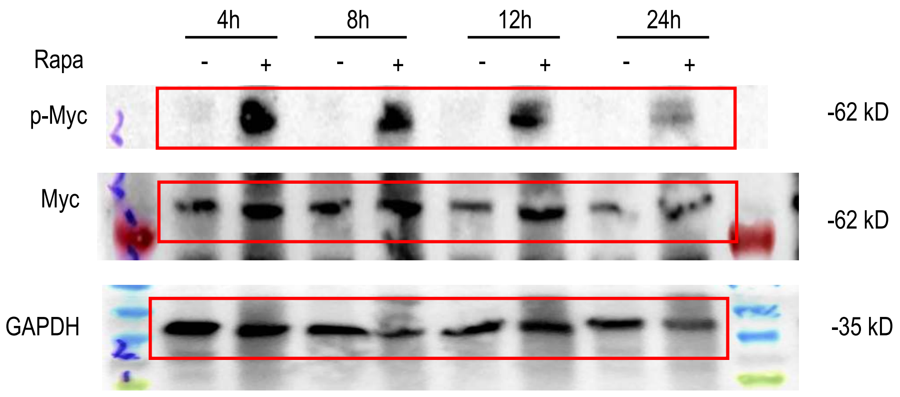

Uncropped blots for Figure 6C

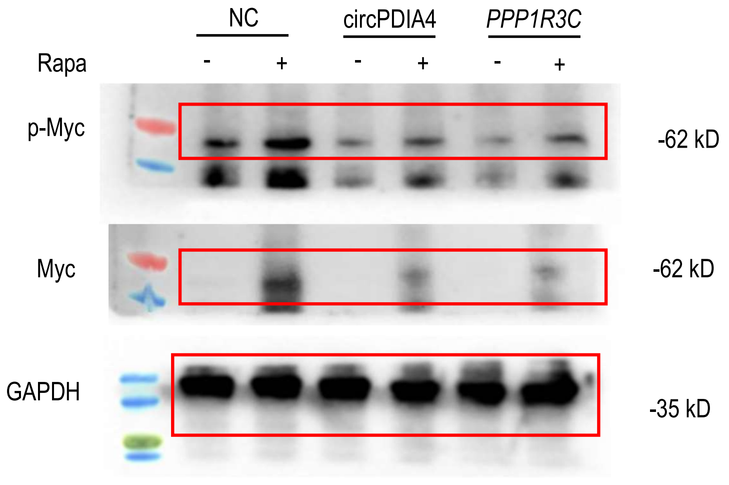

File S2

Uncropped blots for Figure 7B

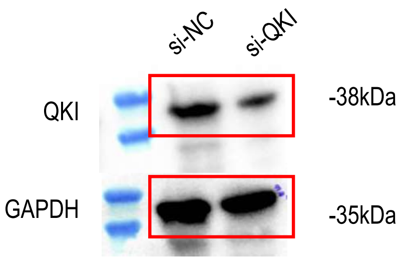

Uncropped blots for Figure 7D

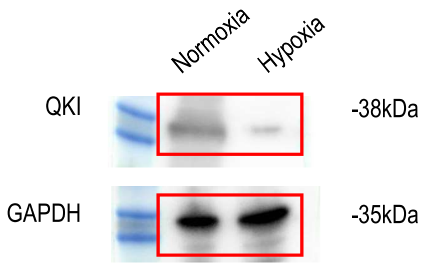

File S2

Uncropped blots for Figure S2G

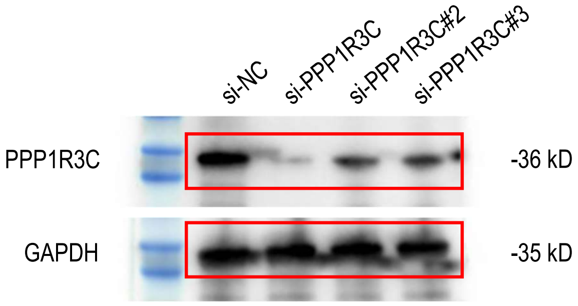

Uncropped blots for Figure S2H

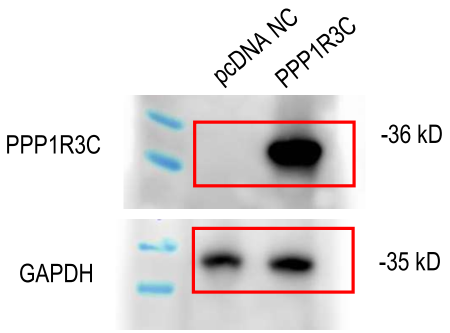

Supplement: Supplementary file 2 — Supplementary Material file 2 [file 41420_2025_2478_MOESM2_ESM.pdf]
